# Eye movement function captured via an electronic tablet informs on cognition and disease severity in Parkinson’s disease

**DOI:** 10.1038/s41598-024-59750-9

**Published:** 2024-04-20

**Authors:** Nils A. Koch, Patrice Voss, J. Miguel Cisneros-Franco, Alexandre Drouin-Picaro, Fama Tounkara, Simon Ducharme, Daniel Guitton, Étienne de Villers-Sidani

**Affiliations:** 1 Integrated Program in Neuroscience, McGill University, Montreal, QC Canada; 2 Montreal Neurological Institute, McGill University, 3801 University Rm 742, Montreal, QC H3A 2B4 Canada; 3 Innodem Neurosciences, Montreal, QC Canada; 4 Douglas Mental Health University Institute, Montreal, QC Canada

**Keywords:** Parkinson's disease, Biomarkers

## Abstract

Studying the oculomotor system provides a unique window to assess brain health and function in various clinical populations. Although the use of detailed oculomotor parameters in clinical research has been limited due to the scalability of the required equipment, the development of novel tablet-based technologies has created opportunities for fast, easy, cost-effective, and reliable eye tracking. Oculomotor measures captured via a mobile tablet-based technology have previously been shown to reliably discriminate between Parkinson’s Disease (PD) patients and healthy controls. Here we further investigate the use of oculomotor measures from tablet-based eye-tracking to inform on various cognitive abilities and disease severity in PD patients. When combined using partial least square regression, the extracted oculomotor parameters can explain up to 71% of the variance in cognitive test scores (e.g. Trail Making Test). Moreover, using a receiver operating characteristics (ROC) analysis we show that eye-tracking parameters can be used in a support vector classifier to discriminate between individuals with mild PD from those with moderate PD (based on UPDRS cut-off scores) with an accuracy of 90%. Taken together, our findings highlight the potential usefulness of mobile tablet-based technology to rapidly scale eye-tracking use and usefulness in both research and clinical settings by informing on disease stage and cognitive outcomes.

## Introduction

Neurodegenerative disorders have long been known to produce a broad variety of oculomotor alterations as a result of deteriorating brain health. Many of these have been previously described in Parkinson's disease (PD) and include, but are not limited to, increased pro-saccade latency^1^, presence of multistep pro-saccades^2,3^, increased saccadic intrusions during fixation^4^, and increased antisaccade error rate^1,5^. Although primarily referred to as a motor neurodegenerative disorder, PD is a multisystem disorder that leads to several non-motor issues, including cognitive dysfunction, dementia, and depression, that contribute greatly to the overall disease burden^6^.

Cognitive dysfunction is one of the more frequent–up to six times more common in individuals with PD than in the healthy population^7^ –and debilitating non-motor symptoms of PD, as it significantly affects the patient’s quality of life^8^. Although it was traditionally believed that cognitive dysfunction does not emerge until the later stages of PD, recent evidence suggests that mild-to-moderate cognitive impairments are often present during the early disease stages, occurring in up to 35% of individuals with early-stage PD^9^. In fact, the onset of cognitive decline appears to be highly unpredictable in PD individuals, which can occur a few years or decades after diagnosis as much as it can appear at the time of, or even prior to, PD diagnosis^10^.

The accurate diagnosis of cognitive impairment in individuals with PD is important for clinical management, and research, including trial selection. Although screening of cognitive function in patients with PD is not performed regularly, it has been argued that it should be part of routine clinical care^11^. The Montreal Cognitive Assessment (MoCA) is the most frequently used cognitive screening instrument in PD research and clinical practice, and the optimal cut-off point of 23/24 has a sensitivity of 0.41 and a specificity of 0.82, with 68% correct diagnoses of PD-MCI^12^. The main drawbacks of such a cognitive screening approach is the limited information gleaned about the detailed cognitive profile and the reduced reliability compared with a comprehensive neuropsychological assessment. However, performing a full neuropsychological assessment is generally too time-consuming to become part of the clinical practice standard of care. Inferring cognitive ability from analysis of oculomotor parameters shows great potential and promise in bridging this gap.

Indeed, a growing body of evidence suggests that eye-tracking data can serve as a viable marker of cognition and cognitive impairments^13,14^. Specifically, several oculomotor metrics measured in individuals with PD have been shown to correlate with measures of general cognition such as the Mini-Mental Status Exam (MMSE)^15,16^ or the MoCA^17,18^. More recently, in a study of individuals with multiple sclerosis, we showed that several oculomotor parameters, when jointly considered, could account for a large proportion of the variance in cognitive test scores^19^.

Despite the promise of oculomotor analysis as a potential marker of cognition and disease severity, this has not previously been practical or scalable given the costs and operational limitations of the required equipment, such as infrared eye-tracking cameras. These limitations acted as important barriers to adoption of eye tracking in clinical practice. To address this critical technological gap, a novel gaze-tracking tool was recently developed and requires only the embedded camera of an iPad Pro (Eye-Tracking Neurological Assessment (ETNA™); Innodem Neurosciences). This approach allows for the precise quantification of several eye movement parameters with a precision comparable to those of research-grade infrared eye tracking devices, such as the latency, velocity, accuracy of saccades, and the presence of saccadic intrusions during fixation. Using this novel technology, we recently replicated sets of well-known oculomotor findings in both individuals with MS^19^ and PD^20^, with the latter study having primarily focused on distinguishing individuals with PD from healthy controls on the basis of recorded eye movement parameters. The main objective of the present paper was to determine to what extent the oculomotor parameters extracted by this mobile eye-tracking tool could serve as viable markers of both disease stage (or severity) using standard PD clinical staging tools, and of cognition in individuals with PD. To address the question of cognition, we evaluated four of the cognitive domains outlined in the Movement Disorder Society Task Force Guidelines^21^ – using one cognitive test per cognitive domain: MoCA (global cognitive), Trail Making Test (attention and working memory), Controlled Oral Word Association Test (COWAT) of verbal fluency (executive function), Hopkins Verbal Learning Test (HVLT; memory). Oculomotor parameters were measured during 5 visual tests that are typically used to reveal eye movement anomalies in various patient populations such as PD^1,4,5,17^: fixation task, pro-saccade task, anti-saccade task, smooth pursuit task, and optokinetic nystagmus task.

In a first preliminary step, we investigated correlations between each cognitive/motor outcome measure of interest and all individual eye movement parameters. We hypothesized based on the known literature that several of these correlations would be of moderate strength (0.3 < r < 0.5), particularly for pro- and anti-saccade parameters. In a subsequent step, we used partial least squares (PLS) regression approaches to determine the extent of clinical score variance that could be explained using the eye movement features and hypothesized that although significant proportions of the variance of the cognitive test scores could be explained, that these proportions wouldn’t be as high as those observed for clinical motor scale scores, as we have previously shown in a sample of patients with MS^19^. Finally, we developed a support vector classifier to discriminate between individuals with mild PD from those with moderate PD (based on UPDRS cut-off scores). Given the strong relationship known to exist between several oculomotor parameters and the UPDRS scores and our own previously published data^20^, we hypothesized that we should be able to distinguish between both PD patient subgroups with a high level of accuracy. The overall aim of the study is to generate evidence that oculomotor parameters collected with a novel tablet-based technology can assist in clinical assessment and management of PD patients by informing on disease severity and cognitive abilities.

## Methods

### Study design and subject population

Patients with mild-to-moderate idiopathic PD were enrolled as a part of the Quebec Parkinson Network (QPN; https://rpq-qpn.ca/) initiative^22^, which includes extensive clinical, neuroimaging, neuropsychological, and biological profiling of participants. A final sample of 65 consecutively recruited patients (age 64.14 ± 8.40, range 45–89, 43/22 males/females) that consented to also undergo eye-tracking experiments were included in this study. No recruited patients were excluded from the dataset. All patients were diagnosed by a movement disorder specialist in the province of Quebec according to the MDS criteria or previously published criteria for patients who were recruited before the publication of the MDS criteria^23^ . All patients with PD were prescribed a stable dosage of antiparkinsonian medication with satisfactory clinical response prior to study enrollment. Patients were instructed to take their medication as prescribed before research visits, and thus all data were collected in the practically-defined “ON” state. Inclusion criteria were confirmed diagnosis of PD and sufficient corrected visual acuity to allow for the accurate reading of the on-screen visual task instructions (see *Gaze-tracking experimental setup* below). Exclusion criteria included comorbid neurological or psychiatric conditions to avoid eye movement anomaly confounders. All participants provided written informed consent. This study was approved by and performed in accordance with the guidelines of the McGill University Health Centre Research Ethics Board Montreal Neurological Institute.

### Clinical and cognitive assessments

To assess clinical status, all PD patients underwent the MDS-UPDRS^24,25^, which was developed to evaluate various aspects of Parkinson’s Disease. Within the context of this study, only the UPDRS full score and the motor subscale (UPDRS part III) were used in data analyses. Patient staging was further performed with the Hoehn and Yahr Scale^26^. Cognitive assessments included the following: the MoCA^27^, the TMT A/B^28^, the HVLT^29^ (immediate recall only), and the COWAT-CFL^30^.

### Gaze-tracking experimental setup

All eye-tracking tests were performed using a 12.9-inch iPad Pro tablet with the ETNA™ software installed, with which it is possible to simultaneously present visual stimuli on-screen and acquire video recordings of the eyes using the embedded front-facing camera at 60 frames per second. Gaze-tracking is performed in visible light with a deep neural network using four inputs and that produces a general gaze model: an image of the user's face, an image of each of the user's eyes, and the Euler angles of the head as head pose information. Apple's ARKit was used to detect facial landmarks.

Prior to undertaking the visual tasks, all participants performed a brief calibration step whereby they track a slow-moving target on-screen. The calibration procedure itself trains an additional model, which is then incorporated into the general gaze model to produce the final individualized gaze-tracking model. The ETNA™ software’s gaze-tracking algorithms have an estimated average (over the entire screen) accuracy of 0.47 degrees (mean offset between the actual gaze position and the recorded gaze position) and precision of 0.33 degrees (as calculated via Root Mean Square (RMS) of the sampled points; an estimate of reliability of the gaze point estimate from one sample to the next, which are comparable values to those of research-grade infrared eye tracking devices.

All participants performed five oculomotor tasks in the predetermined following order: a fixation task, a pro-saccade task, an anti-saccade task, a smooth pursuit task and an optokinetic nystagmus (OKN) task (see below for a more detailed description of each task and Supp. Figure 1 for a visual task representations). All tasks and the calibration step were performed with the tablet screen placed vertically, camera side up, and secured at eye level using a tablet pole mount. The tablet was positioned approximately 45 cm in front of the participants, who were allowed to use their best-corrected visions, with glasses or lenses if necessary. Those who wore corrective eyewear were required to wear them for the full duration of testing, including the calibration step. All participants were instructed to remain still as possible and to neither move their head nor torso for the duration of the eye-tracking tasks. No chin guard or movement restricting device was used to collect data in both a more user-friendly and ecological setting that would more closely mimic at-home testing. Safeguards within the gaze-tracking software ensured the participant’s head was properly positioned in front of the camera and that the eyes were visible, at an acceptable angle and distance from the screen. Safeguards were also in place to ensure lighting conditions were within minimal and maximal thresholds. In the event that a patient fell out of alignment during a task, the application would stop the task and the patient would be required to start that specific task over again. The calibration step and all five tasks were completed in under 15 min.

#### Fixation task

Participants had to fixate a stationary target for 7 s, presented sequentially at five different locations (one central and 4 eccentric locations). The eccentric positions were located 10 degrees of visual angle left and right from the center and 14 degrees of visual angle up and down from the center (Supp. Figure 1**a**).

#### Pro-saccade task

Participants had to initially fixate a central fixation cross, which disappeared after a random period of 1.0–3.5 s, after which a different target reappeared elsewhere on the screen at an eccentric location for 1.5 s either to the left or right, above, or below the central fixation point. Participants were instructed to move their gaze as quickly as possible to the new target location. Both Small (5° horizontal, 6^o^ vertical) and Large (10° horizontal, 12° vertical) amplitude eccentric target distances were used. Each target location was sampled 3 times, for a total of 24 trials (Supp. Figure 1**b**).

#### Anti-saccade task

Participants had to initially fixate a central fixation cross, which disappeared after a random period of 1.0–3.5 s, after which a different target reappeared elsewhere on the screen at an eccentric location (10°) either to the left or right from the center. Participants were instructed to move their gaze as quickly as possible in the opposite direction to the new target location. After being displayed for only 100 ms, the target disappeared, and the screen was left blank for a predetermined duration of time. Following the blank screen, a symbol appeared in the opposite location of where the initial stimulus appeared (i.e., where the participant should be looking). This symbol consisted of a white square with an arrow inside oriented in one of 4 random directions: either left, right, up, or down. Three different difficulty levels were used, each comprising 8 trials. In the Slow trials, the blank screen period lasted 1,200 ms and the arrow symbol duration of 400 ms, in the Medium trials the blank screen period lasted 800 ms and the arrow symbol duration of 250 ms, and in the Fast trials the blank screen period lasted 550 ms and the arrow symbol duration of 100 ms. After each trial, a screen was displayed for 5 s prompting the user to answer which symbol they saw by directing their gaze towards the arrow orientation corresponding to what they believe is the correct answer (Supp. Figure 1**c**). This task was inspired by an anti-saccade task used in a previous study^31^, whereby participants could only identify the second symbol had they performed the anti-saccade task correctly (i.e., looked in the opposite direction of the initial target).

#### Smooth pursuit task

Participants here were first required to fixate a central fixation cross of variable duration (1–2 s). Once the fixation cross disappeared a moving target (that could either go up, down, left or right) appeared on screen for which the participants were instructed to follow with their gaze. Step–ramp paradigm of smooth pursuit at constant velocity was used, whereby the initial position of the moving target was positioned offset from the central fixation point, on the opposite side of the motion direction (Supp. Figure 1**d**). For instance, in a trial of rightward smooth pursuit, the motion target would first appear to the left of the central fixation point (i.e., the step) and then moved in the opposite direction (rightward) at a constant velocity (i.e., the ramp). The trial terminated when the target reached the 10° position either left, right, above, or below center. A total of twelve trials were performed, three in each direction, with a target that moved at one of three constant velocities (Slow: 8.65°/s, with a step size of 1.5°) (Medium: 17.1°/s, with a step size of 2.7°) (Fast: 25.9°/s, with a step size of 4.1°) (see also Supp. Figure 1**d**).

#### Optokinetic nystagmus task

Participants were first required to fixate a central cross for three seconds, after which a 100% contrast horizontally moving vertical square wave grating would appear on screen. The grating stimulus was presented full screen with a fundamental spatial frequency of one cycle per 2.5 degrees. Four different direction and velocity combinations were presented to each participant, each preceded by the fixation cross and each last for 15 s: 1) leftward motion at 4 degrees per second, 2) rightward motion at 4 degrees per second, 3) leftward motion at 8 degrees per second, and 4) rightward motion at 8 degrees per second.

#### Oculomotor parameter extraction

Eye movement parameter extraction was performed offline using ETNA™’s automatic proprietary analysis pipeline. Before parameter extraction, all gaze signals were processed and non-saccadic artifacts (e.g., blinks) were removed by the software’s analysis pipeline. Gaze signals were also denoised using Savitzky-Golay filters. An adaptive, velocity-based algorithm was used for saccade detection, based on the work of Schweitzer and Rolfs^31^. Saccade parameters were then obtained by fitting a parametric model for saccadic waveforms^32^, which reproduces the established relationship between peak saccadic angular velocity and saccadic amplitude (i.e., the saccadic main sequence) to the data acquired during the pro-saccade and anti-saccade tasks. This model fitting provides saccade parameters such as the saccade latency, amplitude and peak velocity.

Fixation parameters were averaged across all positions, resulting in a single set of fixation parameters. Pro-saccades parameters were averaged across locations and dimensions, with the exception of the large amplitude downward saccades, which were removed from all analyses. This removal was done because data for these trials was often absent due to difficulty in detecting the eyes caused by the lowering of the eyelids during large downward saccades. This yielded two sets of prosaccade parameters: one for large amplitude saccades (excluding the data from the downward saccade trials) and one for small amplitude saccades. Anti-saccades parameters were averaged across directions (i.e. left and right), resulting in three sets of anti-saccade parameters, one per difficulty level. Smooth pursuit parameters were averaged across target directions for a given target velocity, resulting in three different parameter sets, one for each velocity used. Finally, OKN parameters were averaged across directions resulting in one parameter set per velocity used. The extracted oculomotor parameters included but were not limited to: saccadic intrusion frequency and amplitude (fixation), saccadic latency, velocity and precision (pro-saccades), direction-specific latency and directional success rate (anti-saccades), amplitude of drift and velocity of return saccade (optokinetic nystagmus), pursuit velocity and the quantification (e.g. count and amplitude) of catch-up saccades (smooth pursuit).

### Correlation analyses

For all correlations between eye movement parameters and the clinical outcome measures of interest (UPDRS-III, UPDRS, H&Y, MoCA, TMTA, TMTB, HVLT, and COWAT-CFL) the Spearman’s ρ correlation coefficient was calculated. For the n = 65 sample size, sensitivity analysis conducted in G*Power^33^ revealed that at α = 0.05 the smallest effect size capable of detection at power = 0.8 is ρ = 0.33. Corrected p-values to adjust for the false discovery rate were computed using the Benjamini–Hochberg procedure evaluated at an alpha level of 0.05^34^.

### Partial least square regression analysis

Partial least squares (PLS) regression was used to examine the relationship between the features and each clinical score (UPDRS-III, UPDRS, H&Y, MoCA, TMTA, TMTB, HVLT, and COWAT-CFL). One of the advantages of using PLS over regression analyses is that it accounts for multicollinearity between oculomotor parameters. A multi-step feature selection procedure was used for each model. The first step consisted in a correlation-based feature selection to determine the 20 most correlated features with the clinical score. Subsequently, an exhaustive feature selection procedure was then used to select the parameter set of the final model, which involved sampling all possible combinations of those 20 oculomotor parameters (set sizes from 1 to 20) and subsequent model fitting. Parameters for which more than half of the participants had a structurally missing value were excluded. For each PLS regression model the number of latent variables maximizing the covariance between the independent and dependent variables was selected by minimizing the Bayesian information criterion^35,36^. For each model, standardized regression coefficients were computed by multiplying regression coefficients by the standard deviation of the predictor variable divided by the standard deviation of the dependent variable. The normalized absolute values of the standardized regression coefficients were used as a measure of oculomotor parameter contribution to the model. The coefficient of determination (R2) was used to assess multiple regression performance (both adjusted and non-adjusted values).

### PD severity subgroup classification

A correlation-based feature selection to determine the 20 most correlated features with UPDRS-III used for training and classification. For the classification of mild (0–35; n = 46) and moderate (36–57; n = 12) UPDRS-III scores^37^, a fivefold stratified cross-validated support vector machine classification method with a radial basis function and ridge regression was used. An exhaustive feature selection procedure was used to select the parameter set of the final model using a balanced accuracy score as the selection metric. This procedure involved sampling all possible combinations of the 20 oculomotor parameters most correlated with UPDRS-III (set sizes from 1 to 3) and subsequent model fitting with fivefold cross validation to determine radial basis function kernel (γ) and the strength of regularization (C). Parameter sets with greater than half of patients having at least one missing oculomotor parameter value missing were excluded. The selected model uses three anti-saccade saccade parameters, relating to the time required to reach the target and the performance rate in accurately detecting the target, and one pro-saccade parameter, relating to mean saccadic velocity, eye-movement parameters as inputs, and has γ = 0.00077, C = 1.0. Receiver Operating Characteristics (ROC) analysis and a confusion matrix were used to assess the performance of the classifier. A classifier for mild, moderate and severe PD (UPDRS-III scores 0–35, 36–57, and > 57 respectively) was selected in a similar manner, however with threefold cross-validation as the severe PD group had a sample size of 3. This classifier uses five different parameters as inputs: three anti-saccade ones relating to the performance rate in accurately detecting the target and saccadic latency, and three pro-saccade parameters relating to mean saccade velocity, peak saccadic velocity and saccadic accuracy, and has γ = 0.0001, C = 0.1984.

Data analysis and visualization were performed using scipy 1.11.1, scikit-learn 1.3.0, matplotlib 3.7.1 and seaborn 0.11.2 in Python 3.10.6.

## Results

### Summary clinical statistics

**Figure 1:**
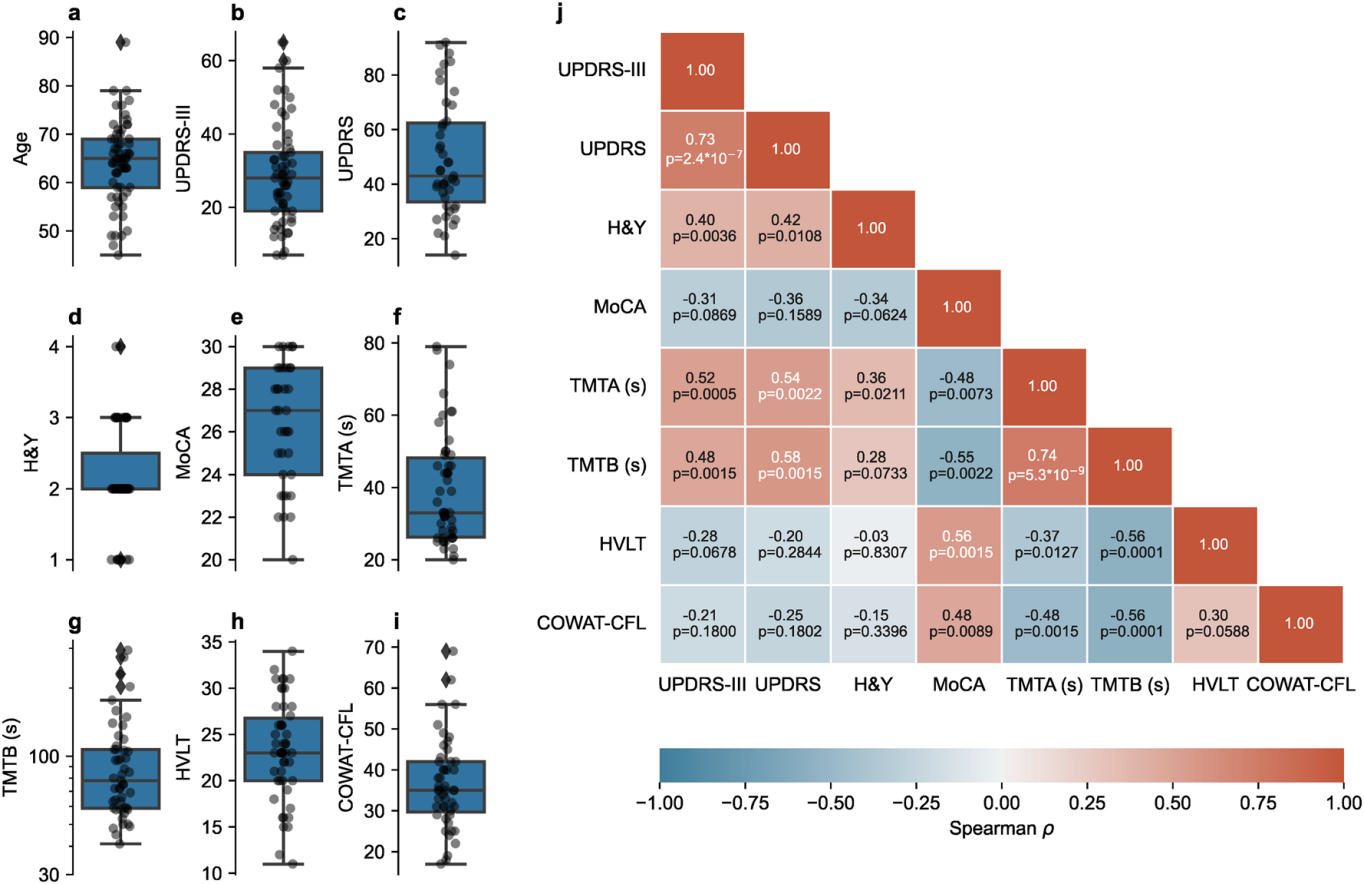
Distribution of participant age (**a**) and participants’ clinical scores: UPDRS-III (**b**), UPDRS (**c**), H&Y (**d**), MoCA (**e**), TMTA (**f**), TMTB (**g**), HVLT (**h**), and COWAT-CFL (**i**). Spearman correlations between participants' clinical scores (**j**).

The distributions of participant age, and scales (UPDRS-III, UPDRS, H&Y) and cognitive test scores (MoCA, TMTA, TMTB, HVLT, and COWAT-CFL) are shown in Fig. 1a–h respectively and summary statistics for each score are found in Table 1. We did not find any significant differences between males and females (see Supp. Figure 2 for further details). Also depicted in Fig. 1 are the magnitudes of the correlations between the different clinical scores (see Supp. Table 1 for details). Related test scores unsurprisingly correlated very highly such as the UPDRS-III and UPDRS (Spearman ρ = 0.7284, corrected *p* = 2.44*10^–7^) and TMTA and TMTB (Spearman ρ = 0.7450, corrected *p* = 5.34*10^–9^). In contrast, the motor scores (UPDRS and UPDRS-III) tended to correlate more poorly with the cognitive scores, such as those from the MoCA, the HVLT and the COWAT-CFL.

**Table 1:** Participant demographic data and PD-related clinical and cognitive test scores.

	n	Mean (SD)	Median (IQR)	Min–max
Age	65	64.14 (8.40)	65 (59, 69)	45–89
UPDRS-III	61	28.98 (13.43)	28 (19, 35)	7–65
UPDRS	43	49.42 (21.07)	43 (33.5, 62.5)	14–92
H&Y	59	2.15 (0.69)	2 (2,2.5)	1–4
MoCA	36	26.39 (2.84)	27 (24, 29)	20–30
TMTA (s)	50	39.52 (15.31)	33 (26.25, 48.25)	20–79
TMTB (s)	49	97.49 (56.6)	78 (59,107)	41–294
HVLT	50	23.36 (5.35)	23 (20, 26.75)	11–34
COWAT-CFL	48	36.56 (11.03)	35 (29.75, 42)	17–69

UPDRS (Unified Parkinson’s Disease Rating Scale); H&Y (Hoehn and Yahr Scale); MoCA (Montreal Cognitive Assessment); TMT-A/B (Trail Making Test—A/B); HVLT (Hopkins Verbal Learning test); COWAT (Controlled Oral Word Association Test).

### Correlations of eye-tracking parameter with clinical scores

**Figure 2:**
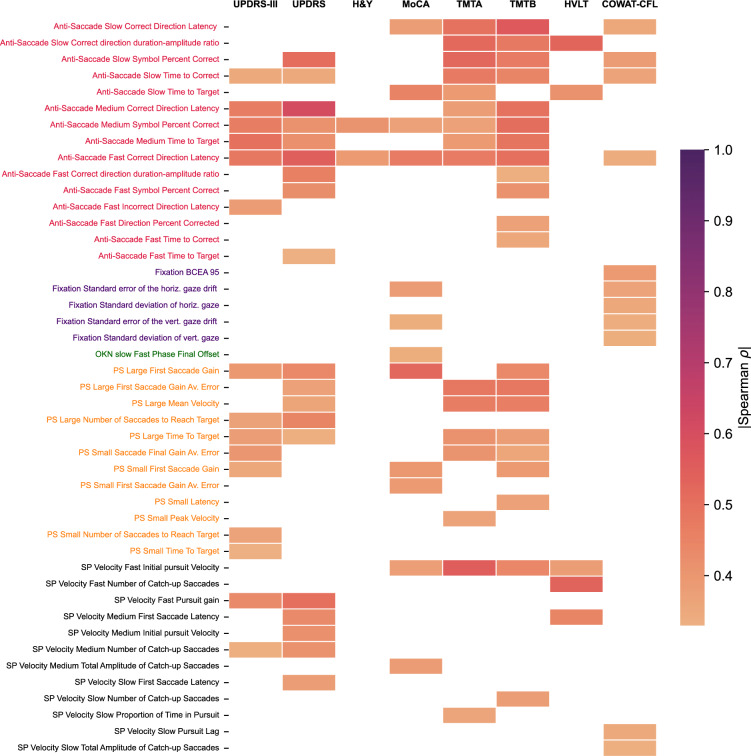
Correlations between eye-tracking parameters and clinical scores. Features with Spearman rho correlation coefficient greater than 0.33 for one clinical outcome measure are shown. Spearman rho correlation coefficient below this threshold of 0.33 are not shown. For a complete correlation matrix see Table 2 and Supplementary Fig. 3. Eye-tracking parameter label colors indicate different oculomotor tasks. PS: pro-saccade, SP: smooth pursuit, OKN: Optokinetic nystagmus.

**Figure 3:**
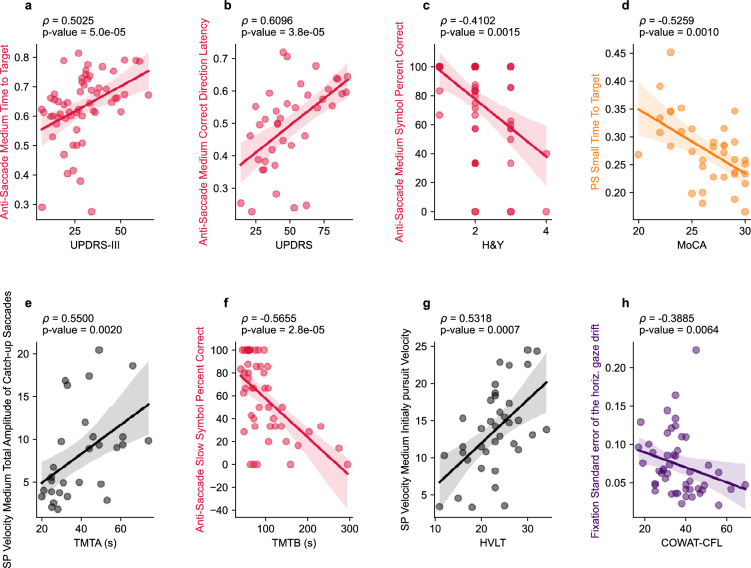
Correlations between select eye-tracking parameters and clinical scores UPDRS-III (**a**), UPDRS (**b**), H&Y (**c**), MoCA (**d**), TMTA (**e**), TMTB (**f**), HVLT (**g**), and COWAT-CFL (**h**). All Spearman's rho correlation values were calculated using the raw data. Linear regressions with 95% confidence intervals are shown for visualization purposes only. PS: pro-saccade, SP: smooth pursuit.

Spearman correlations between the extracted eye movement parameters and clinical outcome measures (UPDRS-III, UPDRS, H&Y, MoCA, TMTA, TMTB, HVLT, COWAT-CFL) are shown in Table 2. The pattern of correlation for each clinical outcome measure is depicted in Fig. 2 after thresholding for correlations with ρ ≥ 0.33 based on the sensitivity analysis for n = 65 sample size at α = 0.05 and power = 0.8. UPDRS and UPDRS-III generally have stronger correlations with eye-movement parameters in the anti-saccade, pro-saccade and smooth pursuit tasks. Amongst those tasks, the parameters that showed the most robust correlations were those relating to movement initiation latency, movement velocity, and movement duration. TMTA and TMTB scores showed very similar correlation patterns to that of UPDRS and UPDRS-III. In contrast, MoCA, HVLT, COWAT-CFL and H&Y exhibited more modest correlations overall compared to the four other tests. H&Y showed similar (but weaker) correlation patterns to the UPDRS and UPDRS-III. After correction for multiple comparisons, 17 eye-movement parameters were significantly correlated with UPDRS-III, 16 eye-movement parameters were significantly correlated with UPDRS, 2 eye-movement parameters were significantly correlated with H&Y, 2 eye-movement parameters were significantly correlated with MoCA, 15 eye-movement parameters were significantly correlated with TMTA, 18 eye-movement parameters were significantly correlated with TMTB, 3 eye-movement parameters were significantly correlated with HVLT, and 8 eye-movement parameters were significantly correlated with COWAT-CFL. Select correlations for each clinical measure are depicted in Fig. 3. The relationships between oculomotor parameters and cognitive scores seen in Fig. 3d and e are further exemplified in Supp. Figure 4, which illustrates how individual pro-saccade and smooth pursuit gaze traces differ between individuals at opposite ends of cognitive score spectrums for the MoCA and the TMTA.

**Table 2:** For each eye-tracking parameter, parameter-clinical score correlations are shown.

Task	Feature	UPDRS-III		H&Y		UPDRS		MoCA		HVLT		TMTA		TMTB *p*-value		COWAT-CFL	
		**ρ**	*p*-value	*p*-value	*p*-value	*p*-value	*p*-value	*p*-value	*p*-value	*p*-value	*p*-value	*p*-value	*p*-value	*p*-value	*p*-value	ρ	*p*-value
Anti-Saccade Slow	Correct Direction Latency	0.216	0.226	0.250	0.217	0.218	0.261	− 0.103	0.627	− 0.202	0.285	**0.524**	**0.001**	**0.470**	**0.004**	**− 0.384**	**0.039**
	Correct direction duration-amplitude ratio	0.214	0.226	0.314	0.164	0.203	0.261	**− 0.454**	**0.043**	**− 0.412**	**0.018**	**0.391**	**0.011**	0.286	0.090	− 0.237	0.243
	Symbol Percent Correct	− 0.280	0.122	− 0.231	0.217	− 0.280	0.115	0.378	0.092	0.287	0.092	**− 0.494**	**0.001**	**− 0.566**	**0.000**	**0.354**	**0.039**
	Incorrect Direction Latency	0.079	0.656	− 0.099	0.655	0.079	0.656	0.319	0.187	0.100	0.599	− 0.150	0.353	0.012	0.941	− 0.191	0.371
	Direction Percent Correct	0.027	0.835	0.049	0.712	0.033	0.800	− 0.107	0.627	0.137	0.465	− 0.217	0.179	− 0.223	0.146	− 0.034	0.821
	Direction Percent Corrected	0.079	0.656	0.172	0.362	0.079	0.656	0.000	0.998	0.007	0.963	− 0.145	0.353	− 0.240	0.146	0.139	0.513
	Time to Correct	0.174	0.453	− 0.083	0.703	0.174	0.453	− 0.295	0.325	**− 0.529**	**0.012**	**0.519**	**0.004**	**0.477**	**0.012**	− 0.114	0.617
	Time to Target	0.346	0.054	0.175	0.362	0.346	0.050	− 0.131	0.627	− 0.325	0.061	**0.472**	**0.002**	**0.448**	**0.004**	**− 0.365**	**0.039**
Anti-Saccade Medium	Correct Direction Latency	**0.464**	**0.001**	0.252	0.175	**0.453**	**0.001**	− 0.239	0.694	− 0.294	0.189	**0.382**	**0.025**	**0.494**	**0.001**	− 0.240	0.250
	Correct direction duration-amplitude ratio	0.047	0.853	0.070	0.834	0.044	0.811	− 0.012	0.947	0.067	0.877	0.153	0.623	0.021	0.890	− 0.045	0.854
	Symbol Percent Correct	**− 0.464**	**0.001**	**− 0.410**	**0.012**	**− 0.470**	**0.001**	0.371	0.248	0.296	0.189	**− 0.371**	**0.025**	**− 0.507**	**0.001**	0.322	0.231
	Incorrect Direction Latency	**0.320**	**0.042**	0.004	0.976	0.303	0.055	0.036	0.947	− 0.175	0.428	0.104	0.817	0.211	0.361	− 0.244	0.250
	Direction Percent Correct	0.024	0.853	− 0.065	0.834	0.031	0.811	− 0.096	0.772	− 0.037	0.902	− 0.053	0.817	− 0.157	0.452	− 0.027	0.854
	Direction Percent Corrected	0.068	0.853	0.038	0.905	0.075	0.811	0.175	0.694	− 0.020	0.902	0.074	0.817	− 0.038	0.890	0.033	0.854
	Time to Correct	− 0.045	0.853	− 0.267	0.176	− 0.050	0.811	0.213	0.694	− 0.244	0.303	0.007	0.970	0.046	0.890	− 0.054	0.854
	Time to Target	**0.502**	**4.0E− 4**	0.247	0.175	**0.488**	**0.001**	− 0.104	0.772	− 0.253	0.220	**0.391**	**0.025**	**0.487**	**0.001**	− 0.240	0.250
Anti-Saccade Fast	Correct Direction Latency	0.163	0.380	0.232	0.287	0.157	0.405	0.124	0.783	− 0.025	0.964	0.086	0.696	0.335	0.056	− 0.238	0.325
	Correct direction duration-amplitude ratio	0.268	0.132	0.031	0.987	0.266	0.133	− 0.134	0.783	0.053	0.964	0.266	0.324	0.052	0.741	0.035	0.946
	Symbol Percent Correct	**− 0.483**	**0.001**	**− 0.388**	**0.028**	**− 0.485**	**0.001**	0.470	0.053	0.306	0.265	**− 0.469**	**0.007**	**− 0.502**	**0.003**	0.343	0.169
	Incorrect Direction Latency	**0.384**	**0.023**	0.037	0.987	**0.382**	**0.023**	− 0.055	0.783	− 0.062	0.964	0.045	0.785	0.123	0.513	0.037	0.946
	Direction Percent Correct	− 0.001	0.992	− 0.063	0.987	0.003	0.982	− 0.079	0.783	0.115	0.964	− 0.162	0.580	− 0.275	0.075	− 0.086	0.894
	Direction Percent Corrected	− 0.089	0.721	− 0.002	0.987	− 0.097	0.663	− 0.069	0.783	0.293	0.265	− 0.115	0.696	**− 0.371**	**0.049**	0.006	0.969
	Time to Correct	− 0.074	0.741	0.018	0.987	− 0.074	0.741	− 0.205	0.783	− 0.074	0.964	0.094	0.696	0.355	0.074	− 0.261	0.325
	Time to Target	0.178	0.370	0.219	0.287	0.169	0.405	0.133	0.783	− 0.007	0.964	0.158	0.580	**0.411**	**0.018**	− 0.212	0.325
Fixation	BCEA 95	0.149	0.433	0.055	0.964	0.144	0.429	− 0.385	0.184	− 0.300	0.094	0.198	0.365	0.221	0.229	**− 0.366**	**0.035**
	Standard error of the horiz. gaze drift	0.255	0.429	0.215	0.458	0.250	0.418	− 0.273	0.273	− 0.260	0.103	0.286	0.178	0.328	0.158	**− 0.388**	**0.035**
	Standard deviation of horiz. gaze	0.184	0.433	0.038	0.964	0.176	0.429	− 0.219	0.273	− 0.290	0.094	0.183	0.365	0.226	0.229	**− 0.356**	**0.035**
	Mean Fixation Duration	− 0.138	0.433	− 0.007	0.964	− 0.138	0.429	0.247	0.273	0.102	0.582	− 0.023	0.986	− 0.082	0.646	− 0.069	0.641
	SI peak velocity	0.161	0.433	− 0.006	0.964	0.158	0.429	− 0.219	0.273	− 0.098	0.582	0.311	0.178	0.238	0.229	− 0.079	0.641
	SI rate	0.063	0.721	0.226	0.458	0.062	0.723	− 0.115	0.503	0.035	0.810	0.002	0.988	0.035	0.811	0.272	0.079
	Percent within 4 degree radius	− 0.215	0.429	− 0.183	0.497	− 0.215	0.418	0.187	0.310	0.261	0.103	− 0.268	0.178	− 0.302	0.158	0.288	0.071
	Standard error of the vert. gaze drift	0.047	0.721	− 0.085	0.964	0.047	0.723	− 0.217	0.273	− 0.322	0.094	0.131	0.541	0.156	0.428	**− 0.336**	**0.035**
	Standard deviation of vert. gaze	0.049	0.721	− 0.018	0.964	0.046	0.723	− 0.337	0.200	− 0.289	0.094	0.116	0.541	0.137	0.448	**− 0.340**	**0.035**
OKN fast	OKN amplitude	− 0.066	0.704	− 0.041	0.947	− 0.060	0.731	0.100	0.867	− 0.021	0.889	− 0.035	0.955	− 0.104	0.789	0.088	0.967
	OKN drift gain	− 0.148	0.466	− 0.228	0.337	− 0.142	0.496	0.093	0.867	0.058	0.772	− 0.102	0.955	− 0.141	0.690	− 0.012	0.967
	Fast Phase Final Offset	0.155	0.466	− 0.038	0.947	0.144	0.496	− 0.215	0.766	− 0.237	0.209	− 0.048	0.955	0.159	0.690	0.068	0.967
	OKN fast phase duration	− 0.193	0.466	0.020	0.947	− 0.195	0.496	0.276	0.599	0.250	0.209	− 0.151	0.955	− 0.281	0.555	0.180	0.967
	OKN frequency	− 0.151	0.466	− 0.263	0.337	− 0.146	0.496	0.110	0.867	0.241	0.209	− 0.017	0.955	− 0.171	0.690	0.006	0.967
OKN slow	OKN amplitude	0.067	0.704	0.238	0.337	0.073	0.731	− 0.036	0.998	− 0.110	0.649	− 0.010	0.955	− 0.072	0.803	0.034	0.967
	OKN drift gain	0.122	0.550	− 0.010	0.947	0.128	0.511	0.000	0.998	− 0.104	0.649	− 0.009	0.955	− 0.035	0.912	0.048	0.967
	Fast Phase Final Offset	0.229	0.466	0.153	0.570	0.219	0.496	− 0.336	0.599	− 0.258	0.209	0.065	0.955	0.092	0.789	0.044	0.967
	OKN fast phase duration	− 0.161	0.466	0.070	0.947	− 0.165	0.496	− 0.002	0.998	0.099	0.649	0.076	0.955	− 0.010	0.951	0.035	0.967
	OKN frequency	− 0.014	0.914	− 0.144	0.570	− 0.012	0.925	0.124	0.867	0.263	0.209	− 0.017	0.955	− 0.184	0.690	0.082	0.967
PS Large	Saccade Final Gain	− 0.270	0.053	− 0.196	0.297	− 0.253	0.071	0.079	0.730	0.032	0.892	− 0.181	0.249	− 0.203	0.194	0.045	0.958
	Saccade Final Gain Av. Error	**0.318**	**0.023**	0.072	0.645	**0.320**	**0.022**	− 0.296	0.292	− 0.158	0.379	0.317	0.075	0.319	0.051	0.038	0.958
	First Saccade Gain	**− 0.378**	**0.014**	− 0.254	0.231	**− 0.357**	**0.022**	0.203	0.409	0.245	0.267	**− 0.413**	**0.014**	**− 0.379**	**0.026**	0.143	0.958
	First Saccade Gain Av. Error	**0.403**	**0.013**	0.154	0.354	**0.401**	**0.022**	− 0.255	0.292	− 0.205	0.267	**0.411**	**0.014**	**0.358**	**0.030**	− 0.091	0.958
	Latency	0.212	0.121	− 0.024	0.856	0.196	0.153	− 0.126	0.640	− 0.087	0.656	0.098	0.529	0.176	0.227	0.015	0.993
	Mean Velocity	**− 0.365**	**0.014**	− 0.224	0.231	**− 0.344**	**0.022**	0.027	0.929	0.223	0.267	− 0.298	0.080	− 0.193	0.207	0.040	0.958
	Peak Velocity	− 0.243	0.082	− 0.132	0.410	− 0.233	0.095	− 0.009	0.959	0.217	0.267	− 0.241	0.127	− 0.308	0.056	− 0.059	0.958
	Number of Saccades to Reach Target	0.219	0.115	0.166	0.340	0.216	0.117	− 0.153	0.559	− 0.309	0.267	**0.366**	**0.033**	0.264	0.110	0.001	0.993
	Time To Target	**0.367**	**0.014**	0.186	0.297	**0.353**	**0.022**	− 0.263	0.292	− 0.272	0.267	0.259	0.113	0.213	0.181	− 0.056	0.958
PS Small	Saccade Final Gain	− 0.172	0.208	− 0.091	0.594	− 0.154	0.261	0.104	0.653	0.120	0.524	− 0.216	0.170	**− 0.330**	**0.047**	0.038	0.958
	Saccade Final Gain Av. Error	**0.317**	**0.023**	0.254	0.231	**0.314**	**0.023**	− 0.247	0.292	− 0.214	0.267	**0.483**	**0.006**	**0.482**	**0.007**	− 0.151	0.958
	First Saccade Gain	− 0.150	0.265	− 0.183	0.297	− 0.132	0.324	0.247	0.292	0.193	0.267	− 0.248	0.124	**− 0.371**	**0.026**	0.068	0.958
	First Saccade Gain Av. Error	**0.330**	**0.021**	0.233	0.231	**0.330**	**0.022**	− 0.284	0.292	− 0.238	0.267	**0.465**	**0.006**	**0.463**	**0.007**	− 0.067	0.958
	Latency	**0.354**	**0.015**	0.068	0.645	**0.335**	**0.022**	− 0.397	0.103	− 0.194	0.267	0.268	0.108	**0.392**	**0.024**	− 0.155	0.958
	Mean Velocity	**− 0.306**	**0.027**	− 0.230	0.231	**− 0.298**	**0.031**	0.197	0.409	0.012	0.933	− 0.073	0.614	− 0.247	0.131	− 0.003	0.993
	Peak Velocity	**− 0.333**	**0.021**	− 0.326	0.212	**− 0.322**	**0.022**	0.114	0.653	− 0.029	0.892	− 0.300	0.080	− 0.180	0.227	− 0.102	0.958
	Number of Saccades to Reach Target	0.088	0.500	0.223	0.231	0.082	0.526	− 0.395	0.103	− 0.202	0.267	0.168	0.273	0.240	0.133	− 0.063	0.958
	Time To Target	**0.399**	**0.013**	0.150	0.354	**0.378**	**0.022**	**− 0.526**	**0.018**	− 0.278	0.267	0.274	0.108	**0.437**	**0.010**	− 0.202	0.958
SP Velocity Fast	First Saccade Latency	0.098	0.830	0.020	0.977	0.100	0.856	− 0.155	0.879	0.161	0.854	− 0.154	0.863	0.155	0.656	− 0.002	0.993
	Initial pursuit Velocity	− 0.191	0.830	− 0.305	0.877	− 0.187	0.856	0.385	0.630	0.260	0.717	− 0.296	0.387	− 0.214	0.570	0.043	0.879
	Number of Catch-up Saccades	− 0.078	0.830	− 0.004	0.977	− 0.078	0.856	− 0.095	0.879	0.194	0.854	− 0.018	0.954	− 0.377	0.279	0.202	0.752
	Pursuit Onset Latency	− 0.102	0.830	− 0.089	0.885	− 0.098	0.856	0.137	0.879	− 0.139	0.854	− 0.069	0.863	0.026	0.919	− 0.061	0.872
	Proportion of Time in Pursuit	0.084	0.830	− 0.138	0.877	0.091	0.856	0.299	0.630	0.033	0.993	− 0.077	0.863	0.280	0.570	− 0.115	0.803
	Pursuit gain	− 0.111	0.830	− 0.254	0.877	− 0.101	0.856	0.226	0.815	0.032	0.993	− 0.360	0.344	− 0.063	0.919	0.033	0.888
	Pursuit Lag	− 0.035	0.855	0.157	0.877	− 0.034	0.856	0.069	0.912	0.019	0.993	− 0.106	0.863	0.047	0.919	− 0.103	0.803
	Total Amplitude of Catch-up Saccades	− 0.168	0.830	− 0.222	0.877	− 0.163	0.856	0.099	0.879	0.142	0.854	− 0.094	0.863	− 0.256	0.570	0.181	0.752
SP Velocity Medium	First Saccade Latency	0.051	0.855	0.166	0.877	0.052	0.856	0.168	0.879	0.040	0.993	− 0.137	0.863	− 0.201	0.656	0.349	0.556
	Initial pursuit Velocity	0.079	0.830	0.102	0.885	0.072	0.856	0.269	0.630	**0.532**	**0.017**	− 0.046	0.939	− 0.212	0.570	0.184	0.752
	Number of Catch-up Saccades	0.120	0.830	0.075	0.885	0.113	0.856	− 0.052	0.921	− 0.025	0.993	0.091	0.863	0.036	0.919	0.335	0.556
	Pursuit Onset Latency	− 0.092	0.830	− 0.157	0.877	− 0.087	0.856	− 0.038	0.921	0.037	0.993	− 0.119	0.863	0.074	0.919	− 0.198	0.752
	Proportion of Time in Pursuit	− 0.067	0.830	− 0.105	0.885	− 0.064	0.856	0.105	0.879	0.114	0.854	− 0.146	0.863	− 0.172	0.656	− 0.114	0.803
	Pursuit gain	0.144	0.830	0.047	0.956	0.149	0.856	0.175	0.879	0.006	0.993	− 0.038	0.939	0.002	0.990	0.064	0.872
	Pursuit Lag	0.062	0.830	0.126	0.885	0.070	0.856	0.042	0.921	− 0.123	0.854	0.026	0.954	0.216	0.570	− 0.206	0.752
	Total Amplitude of Catch-up Saccades	0.040	0.855	0.179	0.877	0.044	0.856	− 0.375	0.630	− 0.382	0.328	0.550	0.048	0.440	0.279	− 0.126	0.803
SP Velocity Slow	First Saccade Latency	− 0.121	0.830	− 0.125	0.885	− 0.091	0.856	− 0.196	0.879	0.002	0.993	− 0.092	0.863	− 0.182	0.656	− 0.132	0.803
	Initial pursuit Velocity	− 0.218	0.830	− 0.155	0.877	− 0.216	0.856	0.010	0.959	0.012	0.993	− 0.295	0.387	− 0.246	0.570	0.242	0.752
	Number of Catch-up Saccades	− 0.334	0.318	0.063	0.914	− 0.334	0.300	− 0.115	0.879	− 0.179	0.854	0.233	0.590	0.251	0.570	− 0.293	0.556
	Pursuit Onset Latency	− 0.021	0.893	0.028	0.977	− 0.021	0.893	− 0.325	0.630	− 0.134	0.854	− 0.010	0.954	0.147	0.656	− 0.115	0.803
	Proportion of Time in Pursuit	0.431	0.085	0.012	0.977	0.428	0.080	0.080	0.912	0.140	0.854	− 0.117	0.863	− 0.136	0.656	0.298	0.556
	Pursuit gain	− 0.062	0.830	0.025	0.977	− 0.057	0.856	− 0.105	0.879	− 0.200	0.854	0.316	0.387	0.168	0.656	− 0.046	0.879
	Pursuit Lag	0.295	0.413	0.078	0.885	0.297	0.380	− 0.281	0.630	− 0.107	0.854	0.259	0.508	− 0.030	0.919	− 0.134	0.803
	Total Amplitude of Catch-up Saccades	0.084	0.830	− 0.177	0.877	0.060	0.856	0.036	0.921	− 0.449	0.328	0.269	0.645	0.062	0.919	0.121	0.803

ρ, Spearman’s rho. *p*-values for two-sided t-tests are reported after FDR correction (Benjamini–Hochberg procedure, α = 0.05). PS: pro-saccade, SP: smooth pursuit, OKN: Optokinetic nystagmus. Correlations with *p* ≤ 0.05 are in bold.

### PLS regression analyses

**Figure 4:**
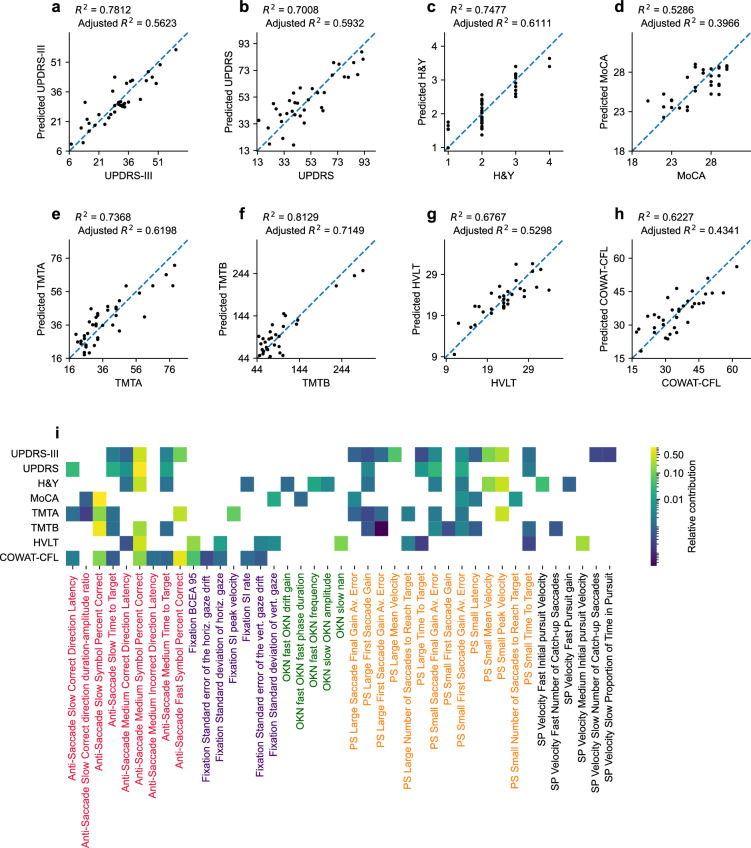
(**a-h**) Scatterplots of the relationship between the study participants’ clinical scores and the corresponding predicted value obtained by partial least squares regression analysis using the oculomotor parameters as predictors. (**i**) Heatmap visualization of the relative contribution (normalized absolute value of standardized regression coefficients) of each oculomotor parameter to each partial least squares regression predictor. Dark squares indicate lesser contributions to the model whereas lighter/yellow squares indicate greater contributions. Absent squares indicate that the parameter was not used in the final model. Eye-tracking parameter label colors indicate different oculomotor tasks. PS: pro-saccade, SP: smooth pursuit, OKN: Optokinetic nystagmus.

To further assess the potential of using oculomotor parameters to estimate clinical outcome indicators, we performed multiple partial least squares (PLS) regression analyses for each clinical outcome measure. Results are presented in Fig. 4a–h and Table 3 and show that all models explain upwards of 56% of the variance of the clinical motor scales (up to 61% for the H&Y scale) and upwards of 40% for the cognitive test scores (up to 71% for TMTB). Specifically, the PLS regression model for UPDRS-III and UPDRS explain 56.23% and 59.32% of the variance in UPDRS-III and UPDRS scores respectively, with important contributions from anti-saccade and pro-saccade eye-movement parameters. The PLS model for H&Y can explain 61.11% of the variance, and has important contributions from anti-saccade, pro-saccade and smooth pursuit eye-movement parameters. In contrast, the MoCA PLS regression model can only explain 39.66% of the variance in MoCA scores with a significant contribution from a single anti-saccade task eye-movement parameter (percentage of trials that participants accurately detected the anti-saccade target, at the easiest difficulty level). The PLS models for the trail making tests explain 61.98% of the variance for TMTA and 71.49% of the variance for TMTB scores, again with anti-saccade and pro-saccade eye-movement parameters the primary contributors. The PLS regression model for HVLT has contributions from a few parameters in every eye movement, which together explain 52.98% of the variance in HLVT scores. The model for COWAT-CFL has significant contributions from anti-saccade and fixation eye-movement parameters and can explain 43.41% of the variance in COWAT-CFL scores. Figure 4i further illustrates the relative contribution of each oculomotor parameter to each model predictor (see also Supp. Table 2). The mean absolute error for the UPDRS and UPDRS-III PLS regressions are 9.66 and 4.37 respectively (Table 3). In general, the inclusion of age as input into the PLS regression does not result in large changes in the amount of variance explained for all models (< 4% except for UPDRS-III (+ 13.76%); Supplementary Fig. 5).

**Table 3:** Mean absolute error (MAE), non-adjusted and adjusted coefficient of determination (R2 and Adj. R2) as well as the F-statistic and associated p-value for the UPDRS-III, UPDRS, H&Y, MoCA, TMTA, TMTB, HVLT, and COWAT-CFL partial least squares regression models in Fig. 4.

	MAE	R^2^	Adj. R^2^		F	*p* value
UPDRS-III	4.372	0.7812	0.5623	F (18, 16)	3.1731	2.97E− 05
UPDRS	9.659	0.7008	0.5932	F (10, 24)	5.6227	1.03E− 06
H&Y	0.313	0.7477	0.6111	F (14, 23)	4.8692	2.91E− 05
MoCA	1.563	0.5286	0.3966	F (8, 24)	3.3635	6.59E− 04
TMTA	6.587	0.7368	0.6198	F (13, 26)	5.5985	6.47E− 06
TMTB	20.720	0.8129	0.7149	F (12, 20)	7.2420	6.23E− 05
HVLT	2.383	0.6767	0.5298	F (11, 21)	3.9968	2.03E− 05
COWAT-CFL	5.408	0.6227	0.4341	F (12, 21)	2.8885	2.71E− 04

### Classification

**Figure 5:**
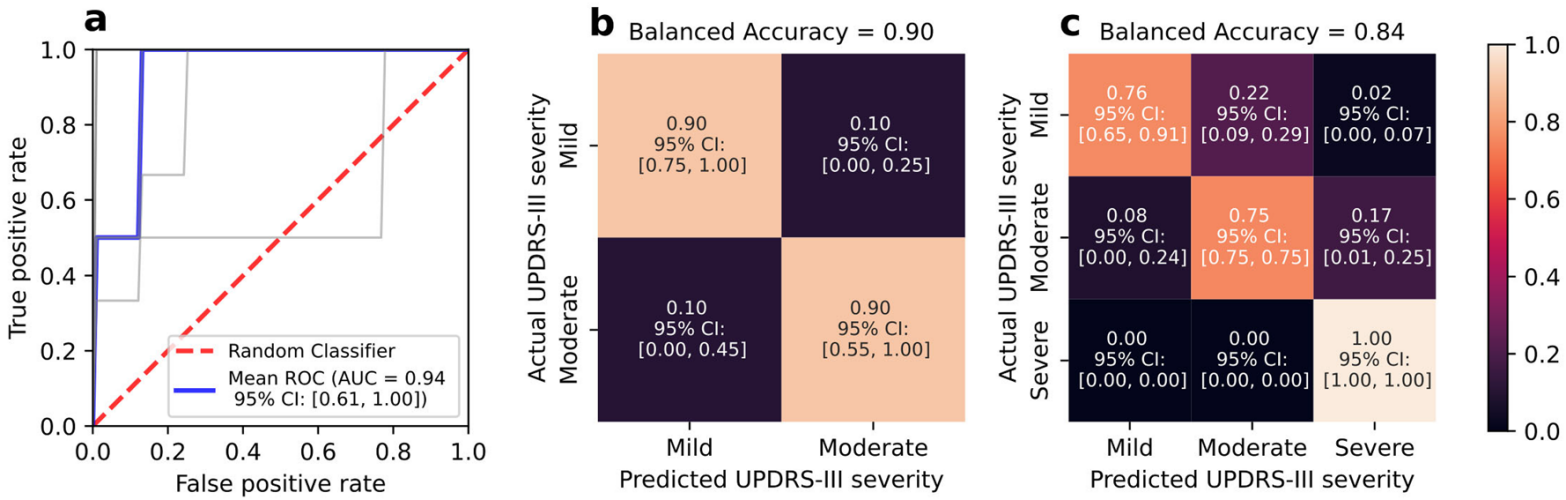
Performance of the support vector classifier. (**a**) Mean ROC curve for the logistic regression classifier across fivefold cross validation. Grey lines represent each fold. (**b**) Confusion matrix for classification of mild (UPDRS-III 0–35) and moderate (UPDRS-III 36–57) Parkinson's Disease using eye tracking parameters. (**c**) Confusion matrix for classification of mild (UPDRS-III 0–35), moderate (UPDRS-III 36–57) and severe (UPDRS-III > 57) Parkinson's Disease using eye tracking parameters.

The average Receiver Operating Characteristic curve for the support vector classifier is computed and the area under the curve (AUC) is 0.94 (95% CI [0.61–1.00]; Fig. 5a). The classifier has a sensitivity of 0.90 (95% CI [0.75, 1.00]) and specificity of 0.90 (95% CI [0.55–1.00]; Fig. 5b) for classifying mild PD from moderate PD on the basis of UPDRS-III scores (0–35 and 36–57, respectively). The classifier for mild, moderate and severe PD (UPDRS-III scores 0–35, 36–57, and > 57 respectively) has a balanced accuracy of 0.84 with accuracy of 0.76 for mild, 0.75 for moderate and 1.0 for severe PD (Fig. 5c). Overall classifier performance does not change for either classification with the inclusion of age (Supplementary Fig. 6).

## Discussion

The present study provides several clinically relevant lines of evidence supporting the usefulness of monitoring the oculomotor system as a means to assess both disease severity and cognitive function in PD. First, our findings confirm those from our previous study^20^ using the same technology with PD patients, that several oculomotor parameters strongly correlated with measures of disease stage and severity such as the UPDRS-III score. Second, in line with previous publications in the literature we provide confirmatory evidence that individual oculomotor motor parameters significantly correlate with various measures of cognitive function in individuals with PD. Third, using regression analysis approaches that were implemented in a recent analysis of the same technology in a cohort of multiple sclerosis patients^19^, we show that combining the information of several oculomotor parameters can explain upwards of 56% of the variance of the clinical motor scales (up to 61% for the H&Y scale) and upwards of 40% for the cognitive test scores (up to 71% for TMTB). Finally, we show that all of these findings can be obtained using a novel mobile tablet-based eye tracking system, which has the potential to both optimize clinical care of patients with PD, as well as accelerate eye movement research by being both an affordable and scalable solution to help characterize disease status, monitor disease progression, and track changes in cognitive ability.

With our previous study^20^ having primarily focused on distinguishing individuals with PD from healthy controls on the basis of recorded eye movement parameters, one of the main objectives of the present paper was to determine to what extent the oculomotor parameters extracted by this mobile eye-tracking tool could serve as a viable marker of cognition in individuals with PD. More specifically, here we investigated the extent to which oculomotor parameters could be used to infer cognitive ability as measured by specific neuropsychological tests.

### Link between oculomotor parameters and cognitive domains

Although there is ample evidence demonstrating the existence of alterations of eye movements (and associated oculomotor parameters) in PD patients^1,2,4,38,39^, a smaller body of work points to relationships between some of these parameters and cognitive test scores. For instance, several parameters of fixation stability were shown to correlate with MMSE test scores^16^, a test of cognitive screening similar to the MoCA. MoCA test scores have been shown to correlate with pro-saccade parameters of latency^17,39^ and accuracy^17^, and anti-saccade latency^1^. In a sample of essential tremor patients, the presence of pro-saccade, anti-saccade and smooth pursuit impairments (relative to healthy controls) strongly correlated with low MMSE scores and impaired verbal fluency^40^. Similarly, van Stockum et al.^41^ identified a link between verbal fluency ability and anti-saccade latency in individuals with PD.

To determine to what extent the oculomotor parameters when combined could serve as a viable marker of cognition in individuals with PD, we used a partial least squares (PLS) regression, which accounts for multicollinearity among the predictor variables (oculomotor parameters). This approach yielded adjusted R-squared values between 0.3966 (MoCA) and 0.7149 (TMTB). On the whole, adjusted R-squared values were lower for measures of general cognition (MoCA), verbal fluency (COWAT-CFL) and verbal memory (HVLT) (between 0.4341 and 0.5298). That we were able to explain less of the observed variance in these tests is perhaps not surprising given that the MoCA is a multi-domain metric and both the HVLT (verbal memory) and COWAT-CFL (verbal fluency) tap into cognitive processes that have less obvious links to the oculomotor system, which is primarily assessed via eye movement parameters of latency, velocity and duration. Interestingly, the MoCA and the HVLT tasks were those whose PLS models had a more even distribution of contributions from the different eye-movement tasks (as opposed to a strong bias towards pro-saccade and anti-saccade tasks as for most of the other clinical score scales). This finding further highlights the multi-domain nature of these cognitive tests.

In contrast, higher adjusted R-squared values for the UPDRS and UPDRS-III (0.5623 and 0.5932, respectively) were expected given that their measures are strongly biased towards motor impairments (particularly for the UPDRS-III). As seen in Fig. 4, there is a far greater number of contributing parameters to the PLS models from the anti-saccade and pro-saccade tasks. In fact, the best models for UPDRS and UPDRS-III are almost exclusively composed of parameters from the two saccade tasks. For the anti-saccade task, almost all the contributing parameters were related to timing, latency and duration of saccadic eye movements and processes. For the pro-saccade task, the contributing factors were a mix of timing, accuracy and velocity parameters. The findings are in line with the observation that PD significantly affects the timing and accuracy of movements in general^42^.

Indeed, although the high adjusted R-squared values for the TMTA and TMTB tasks (0.6198 and 0.7149, respectively) were less expected initially, upon closer investigation they were not without precedent from neither a cognitive perspective nor a motor perspective. For instance, the vast majority of contributing parameters to the TMTA and TMTB models were parameters from the anti-saccade and pro-saccade tasks (see Fig. 4, panel i), which, in fact, significantly overlapped with the contributing factors for UPDRS/UPDRS-III models. This finding is less surprising when looking at the correlation matrix in Fig. 1**,** which illustrates that both TMTA and TMTB scores strongly correlate with UPDRS/UPDRS-III scores (all r > 0.48). In fact, those correlation coefficients with UPDRS/UPDRS-III are not only higher than for any other cognitive test but are also higher than for the H&Y stage. The relationship between UPDRS/UPDRS-III and TMTA/TMTB likely stems from the important psychomotor execution and planning components required to adequately perform the trail making tests.

From a cognitive perspective, anti-saccade parameters, such as those related to error rate and reaction time, have been shown in the past to correlate with several measures of executive function, particularly on tasks that involve psychomotor speed, visual search, attention task-switching, and inhibition, such as the TMTA and TMTB tests^43,44^, supporting the notion that the anti-saccade task taps into several executive and frontal cognitive processes, including but not limited to cognition inhibition^45–47^. Further supporting this hypothesis of tapping into multiple cognitive processes is the fact that the anti-saccade task is the only one of the five for which parameters contribute to every cognitive test PLS model, highlighting the potential usefulness of anti-saccade task parameters to serve as a marker for several cognitive processes.

These saccadic findings as a whole are also consistent with the findings of a recent study that used a cluster analysis to identify different patterns in saccade abnormalities in PD and their relationship with cognitive phenotypes^48^. The authors identified three clusters, one of which was defined by a general disinhibition of reflexive saccades (i.e. antisaccade errors) and executive dysfunction. A second cluster consisted of individuals with multi-domain impairment accompanied by high antisaccade error rates, prolonged saccadic latencies and pronounced saccadic hypometria, consistent with our findings that these parameters were found to contribute to the explanatory models of several cognitive tests.

### Group classifications based on oculomotor parameters

Using a support vector classifier, we were able to separate individuals with mild PD from those with moderate PD (determined on the basis of their UPDRS-III scores) using oculomotor parameters with a sensitivity of 0.90 and specificity of 0.90, and an AUC of 0.94. Mild, moderate, and severe PD (likewise based on UPDRS-III scores) could be classified with an accuracy of 0.84. The classifier performances are comparable to that of a recent study that used saccade, blink and pupil parameters to separate PD patient subgroups with distinct cognitive deficits (e.g. PDD, PD-MCI) from healthy controls^49^. They found an overall AUC of 0.88 (sensitivity of 83% and a specificity of 78%), with the subgroups showing a progressive increase of the AUC with progressing cognitive deficits and the AUC of PDD alone being 0.95. Although we did not include healthy controls in our analysis, the fact that we were able to separate subsets of PD patients from one another based on disability suggests that a classifier with a high accuracy could have been built with healthy controls included as well. However, our goal here was not to evaluate to what extent we could detect PD in an individual, but rather if we could identify oculomotor parameters whose values are tied to disease severity, with the long-term goal of potentially being able to track progression of both motor and cognitive disability.

Other previous work has also demonstrated the usefulness of eye movement metrics to develop classifiers that can distinguish between PD patients and healthy controls, such as in Tseng et al.^50^ (89.6% accuracy, 85% sensitivity and 93% specificity), Tsitsi et al.^16^ (82% accuracy). Overall, our findings demonstrate that it is possible to develop accurate classifiers that are highly comparable to those developed in prior studies while using parameters captured via an electronic tablet.

### Potential clinical uses for a mobile tablet-based eye-tracking device

The present findings suggest that collecting eye-tracking data with mobile technology has the potential to provide precise information on both the disease stage and cognitive ability of PD patients, which could prove useful to clinicians and researchers alike. We believe the current findings to be a promising first step in the development of a mobile tool that could move beyond correlations and explained variance towards clinical score estimation. As additional data continues to be collected, more sophisticated and multi-layered machine-learning algorithms can be developed to estimate or predict clinical scale or cognitive test scores (within an acceptable range of accuracy) based on the various eye-movement parameters extracted with the mobile gaze-tracking software. Following proper validation studies, we believe such a predictive tool could have a significant impact on clinical management and drug discovery by enabling quick disease staging (< 10 min) without the need for a lengthy clinical examination. Indeed, a full assessment to characterize a PD patient’s cognitive and motor function generally takes upwards of an hour. In addition to potentially aiding and accelerating clinical management, such a tool could be used as a quick pre-screening aid for clinical trial enrollment. It could also serve as disease monitoring aid to assess progression and response to treatment and could even be performed at home—reducing the need for in-clinic visits only when changes are detected in the eye-tracking data. Current ongoing clinical trials in MS patients support the notion that at-home testing using a tablet-based tablet-based eye-tracking device for monitoring progression is not only viable but also clinically useful^51^.

Not all tracking tests used in the present study contribute equally to motor and cognitive score estimation. Indeed, anti-saccade and pro-saccade parameters were the largest contributors to most PLS models. The most relevant pro-saccades parameters were those related to saccade latency or duration, velocity, and accuracy, whereas the most relevant anti-saccade parameters were those related to latency and timing, correctly performing the anti-saccade trial and correctly identifying the final target. Overall, our data suggest that it may be possible to remove some eye-tracking tasks with little penalty in terms of estimation accuracy, depending on the desired output metrics, while significantly cutting down on the time required to conduct the eye-tracking testing (which already only takes 10–15 min). In particular, the smooth pursuit and OKN tasks did not contribute significantly to the different PLS models used.

### Study limitations

One potential study limitation is that we limited the patient sample to PD patients. Current evidence suggests that related parkinsonion syndromes tend to exhibit certain eye movement differences with PD patients, and this appears to be particularly true of individuals with inherited parkinsonisms^52^, PSP-parkinsonism^53^, Parkison’s disease dementia (PDD) and dementia with Lewy bodies (DLB)^54^. Future studies could benefit from the inclusion of other parkinsonian phenotypes to increase the generalizability of the findings. Along the same line, our study sample was primarily composed of individuals situated on the mild-to-moderate spectrum of the disease (based on their UPDRS-III scores and the H&Y stages), thus it remains unclear to what extent the oculomotor parameters identified in the present study would enable proper classification of individuals with more advanced PD. Finally, although we did investigate some cognitive domains, not all of them were sampled and stronger relationships with oculomotor parameters could have been observed with neuropsychological tests that are not investigated in this present study.

Among the present study limitations is the small sample size, which was only powered to detect significant correlations for correlation coefficients above rho = 0.33. Nonetheless, we were still able to identify numerous correlations that survived multiple comparison corrections. The sample size is also a limitation of the group classification analysis, as the small sample size gives rise to a heightened risk of overfitting, however the use of fivefold (and threefold) stratified cross-validation are used to minimize this risk and to maximize generalizability. The inclusion of the small sample size (n = 3) of severe PD patients decreases the performance of the classifier from a balanced accuracy of 0.9 to 0.84, in large part due to data imbalance that may be rectified by larger samples of severe patients and a more balanced dataset. Although here we show very promising results via the partial least squares regression analyses that suggest that it may be possible to do so, such analyses produce inference models, which do not guarantee strong predictive abilities. Indeed, to be able to confidently claim that we can estimate disease cognitive ability in a single individual, predictive models would need to be validated with an independent dataset.

Another limitation of the present study is that all data were gathered in supervised settings. Although, as we mentioned above, there is evidence from an ongoing clinical trial in MS patients using the same device that suggests the data collected at home is equivalent to that collected in supervised settings, this has yet to be quantitatively analyzed and published in a peer-reviewed journal. Finally, another limitation relates to the camera position relative to the tablet, which leads to difficulty in accurately detecting the eyes when they are looking at the bottom of the screen. As a result, we had to remove data collected from large amplitude downward saccades from all analyses.

### Conclusion

The present findings support the general view that in-depth oculomotor examination can be of clinical use for a variety of disorders^55^. More specifically, we have shown that when several key relevant oculomotor parameters are combined together, they can explain large proportions of cognitive test score variance in PD patients. Moreover, we provide evidence that these eye-tracking data can be leveraged to build classifiers that can reliably discriminate between population sets, in this case patients with *mild* PD patients and those with *moderate* PD. Taken together, these findings suggest that with continued data collection and algorithmic refinement, tablet-based eye-tracking tools have the potential to at the very least become a critical part of the standard clinical assessment of various patient populations, if not one day replace some of the current standard of care practices that are often more time consuming and less objective.

### Supplementary Information

Supplementary Information.

## Data Availability

The datasets used and/or analyzed during the current study are available from the corresponding author on reasonable request.

## Code availability

The underlying code for this study [and training/validation datasets] is not publicly available but may be made available to qualified researchers on reasonable request from the corresponding author.
