# Supplementary material for: Eye movement function captured via an electronic tablet informs on cognition and disease severity in Parkinson’s disease
Source: Sci Rep. 2024 Apr 20;14:9082. doi: 10.1038/s41598-024-59750-9 (PMC11032372; doi:10.1038/s41598-024-59750-9)
Supplement: Supplementary file 1 — Supplementary Information. [file 41598_2024_59750_MOESM1_ESM.pdf]

Supplementary Material

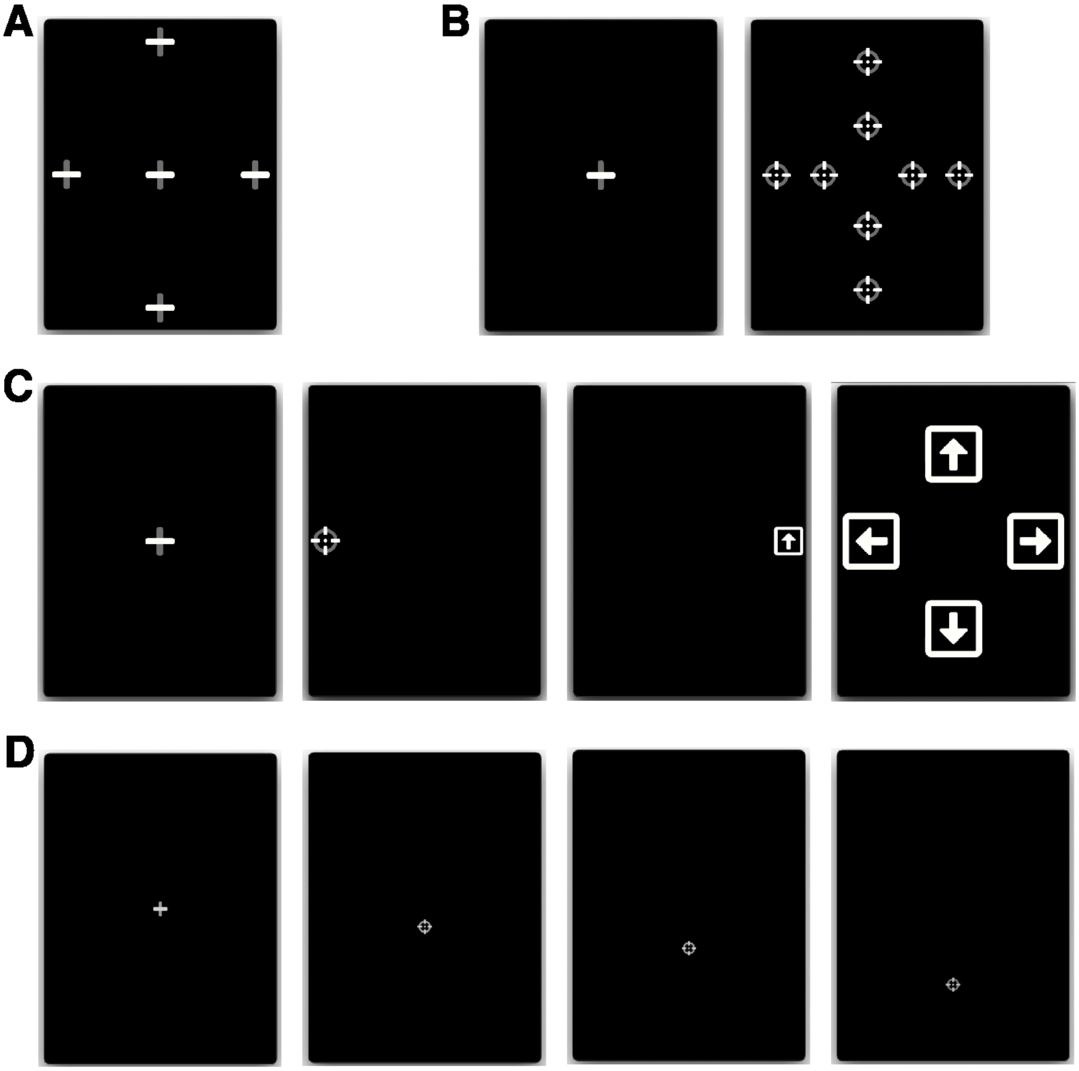

Supplementary Figure 1. Eye-tracking tasks. **(A)** Fixation: participants fixated a stationary target for 7 seconds, at one of 5 locations. **(B)** Pro-saccades: participants initially fixated a central fixation point, which disappeared after 1.0 – 3.5 s, after which a different target appeared at one of 8 eccentric locations for 1.5 seconds. **(C)** Anti-saccades: participants initially fixated a central fixation point, which disappeared after 1.0 – 3.5 s, after which a round target appeared at 10° to the left or right from the center. Participants were instructed to move their gaze in the opposite direction to the round target, where after 1200ms they were shown a square with an arrow inside that pointed in one of 4 random directions (left, right, up, or down; shown during 400ms). The users then had to direct their gaze toward the arrow orientation corresponding to the arrow they saw in the preceding step. **(D)** Smooth pursuit: after initially fixating on a central cross, participants followed a moving target with a constant velocity of 8°/s (in this example, a downward-moving target).

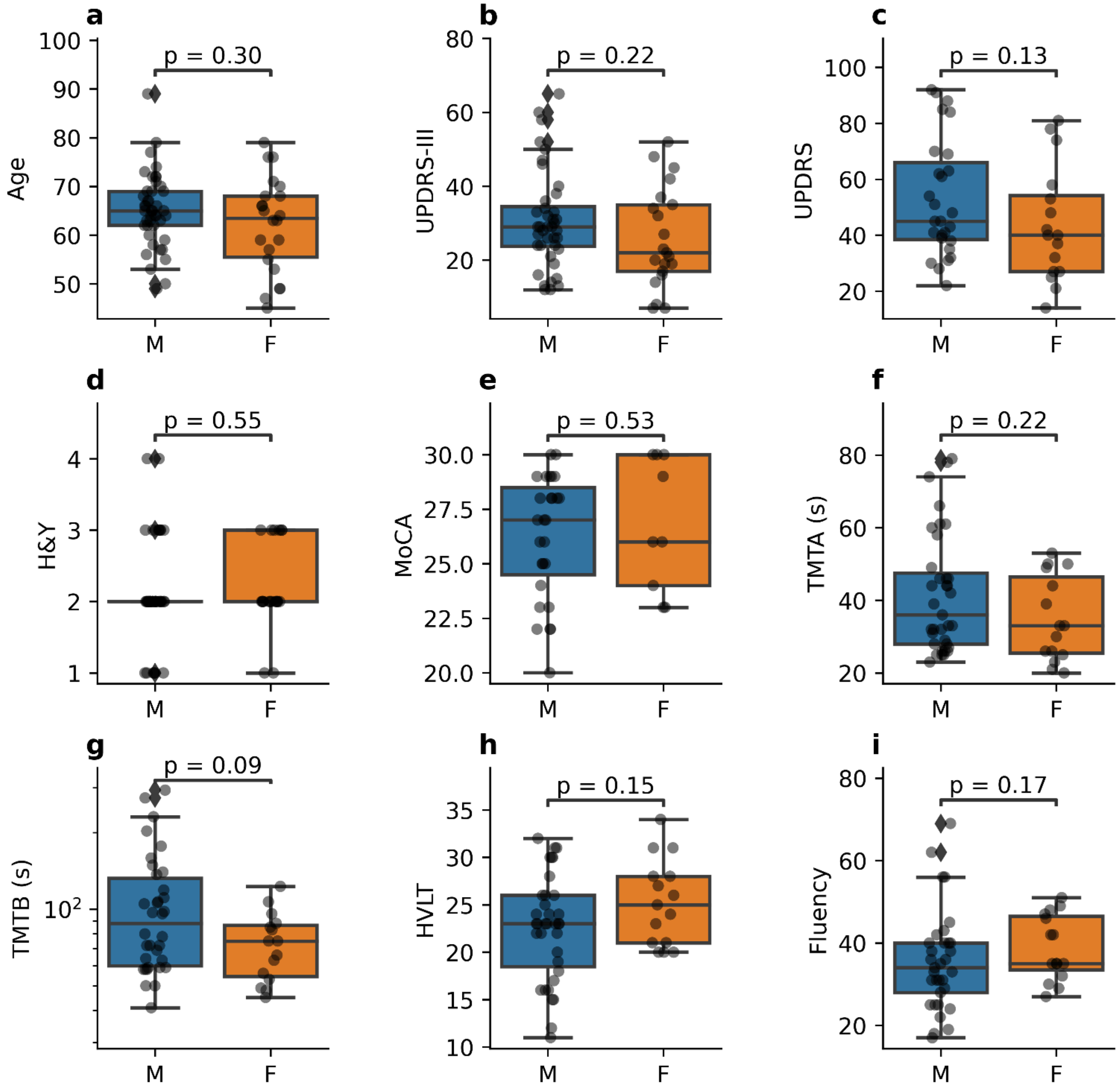

Supplementary Figure 2. Lack of differences between male (M) and female (F) participant age (**a**; Mann–Whitney U = 547.5, n_1 = 43, n_2 = 22, p =0.3043 two-tailed) and participants' clinical scores: UPDRS-III (**b**; Mann–Whitney U = 501.0, n_1 = 40, n_2 = 21, p = 0.2215 two-tailed**)**, UPDRS (**c**; Mann–Whitney U = 277.0, n_1 = 27, n_2 = 16, p = 0.1284 two-tailed, H&Y (**d**; Mann–Whitney U = 357.0, n_1 = 39, n_2 = 20, p = 0.5505 two-tailed, MoCA (**e**; Mann–Whitney U = 104.0, n_1 =27, n_2 = 9, p = 0.5317 two-tailed), TMTA (**f**; Mann–Whitney U = 320.5, n_1 = 35, n_2 = 15, p = 0.2228 two-tailed**)**, TMTB (**g**; Mann–Whitney U = 334.5, n_1 = 34, n_2 = 15, p = 0.0864 two-tailed), HVLT (**h**; Mann–Whitney U = 194.5, n_1 = 35, n_2 = 15, p = 0.1516 two-tailed), and COWAT-CFL (**i**; Mann–Whitney U = 185.5, n_1 = 33, n_2 = 15, p = 0.1707 two-tailed).

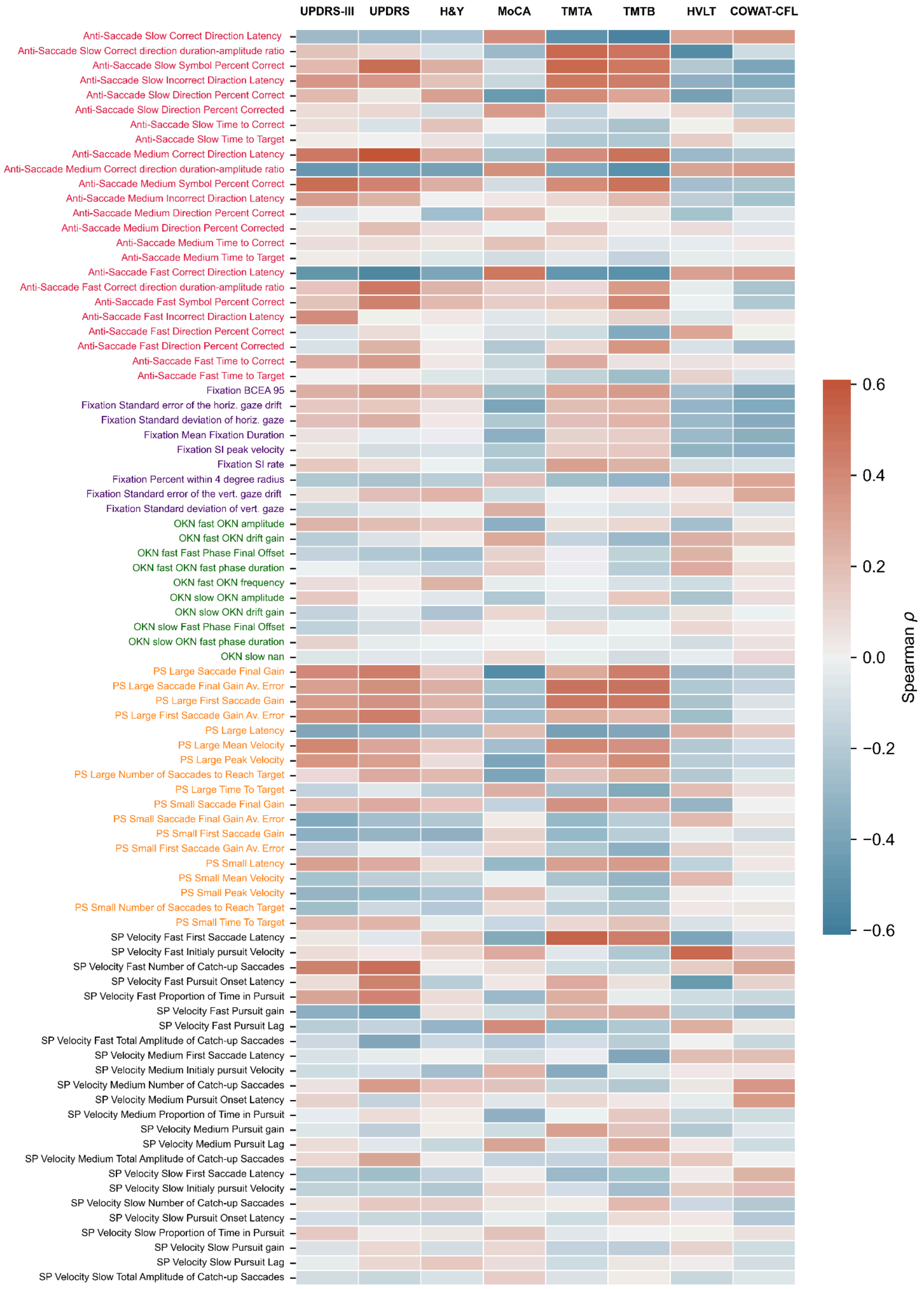
UPDRS-III
UPDRS
H&Y
MoCA
TMTA
TMTB
HVLT
COWAT-CFL
Anti-Saccade Slow Direction Latency
Anti-Saccade Slow Correct direction duration-amplitude ratio
Anti-Saccade Slow Symbol Percent Correct
Anti-Saccade Slow Incorrect Diraction Latency
Anti-Saccade Slow Direction Percent Correct
Anti-Saccade Slow Direction Percent Corrected
Anti-Saccade Slow Time to Correct
Anti-Saccade Slow Time to Target
Anti-Saccade Medium Correct Direction Latency
Anti-Saccade Medium Correct direction duration-amplitude ratio
Anti-Saccade Medium Symbol Percent Correct
Anti-Saccade Medium Incorrect Diraction Latency
Anti-Saccade Medium Direction Percent Correct
Anti-Saccade Medium Direction Percent Corrected
Anti-Saccade Medium Time to Correct
Anti-Saccade Medium Time to Target
Anti-Saccade Fast Correct Direction Latency
Anti-Saccade Fast Correct direction duration-amplitude ratio
Anti-Saccade Fast Symbol Percent Correct
Anti-Saccade Fast Incorrect Diraction Latency
Anti-Saccade Fast Direction Percent Correct
Anti-Saccade Fast Direction Percent Corrected
Anti-Saccade Fast Time to Correct
Anti-Saccade Fast Time to Target
Fixation BCEA 95
Fixation Standard error of the horiz. gaze drift
Fixation Standard deviation of horiz. gaze
Fixation Mean Fixation Duration
Fixation SI peak velocity
Fixation SI rate
Fixation Percent within 4 degree radius
Fixation Standard error of the vert. gaze drift
Fixation Standard deviation of vert. gaze
OKN fast OKN amplitude
OKN fast OKN drift gain
OKN fast Fast Phase Final Offset
OKN fast OKN fast phase duration
OKN fast OKN frequency
OKN slow OKN amplitude
OKN slow OKN drift gain
OKN slow Fast Phase Final Offset
OKN slow OKN fast phase duration
OKN slow nan
PS Large Saccade Final Gain
PS Large Saccade Final Gain Av. Error
PS Large First Saccade Gain
PS Large First Saccade Gain Av. Error
PS Large Latency
PS Large Mean Velocity
PS Large Peak Velocity
PS Large Number of Saccades to Reach Target
PS Large Time To Target
PS Small Saccade Final Gain
PS Small Saccade Final Gain Av. Error
PS Small First Saccade Gain
PS Small First Saccade Gain Av. Error
PS Small Latency
PS Small Mean Velocity
PS Small Peak Velocity
PS Small Number of Saccades to Reach Target
PS Small Time To Target
SP Velocity Fast First Saccade Latency
SP Velocity Fast Initialy pursuit Velocity
SP Velocity Fast Number of Catch-up Saccades
SP Velocity Fast Pursuit Onset Latency
SP Velocity Fast Proportion of Time in Pursuit
SP Velocity Fast Pursuit gain
SP Velocity Fast Pursuit Lag
SP Velocity Fast Total Amplitude of Catch-up Saccades
SP Velocity Medium First Saccade Latency
SP Velocity Medium Initialy pursuit Velocity
SP Velocity Medium Number of Catch-up Saccades
SP Velocity Medium Pursuit Onset Latency
SP Velocity Medium Proportion of Time in Pursuit
SP Velocity Medium Pursuit gain
SP Velocity Medium Pursuit Lag
SP Velocity Medium Total Amplitude of Catch-up Saccades
SP Velocity Slow First Saccade Latency
SP Velocity Slow Initialy pursuit Velocity
SP Velocity Slow Number of Catch-up Saccades
SP Velocity Slow Pursuit Onset Latency
SP Velocity Slow Proportion of Time in Pursuit
SP Velocity Slow Pursuit gain
SP Velocity Slow Pursuit Lag
SP Velocity Slow Total Amplitude of Catch-up Saccades
0.6
0.4
0.2
0.0
−0.2
−0.4
−0.6
Spearman ρ

Supplementary Figure 3. Correlations between eye-tracking parameters and clinical scores. PS: pro-saccade, SP: smooth pursuit, OKN: Optokinetic nystagmus

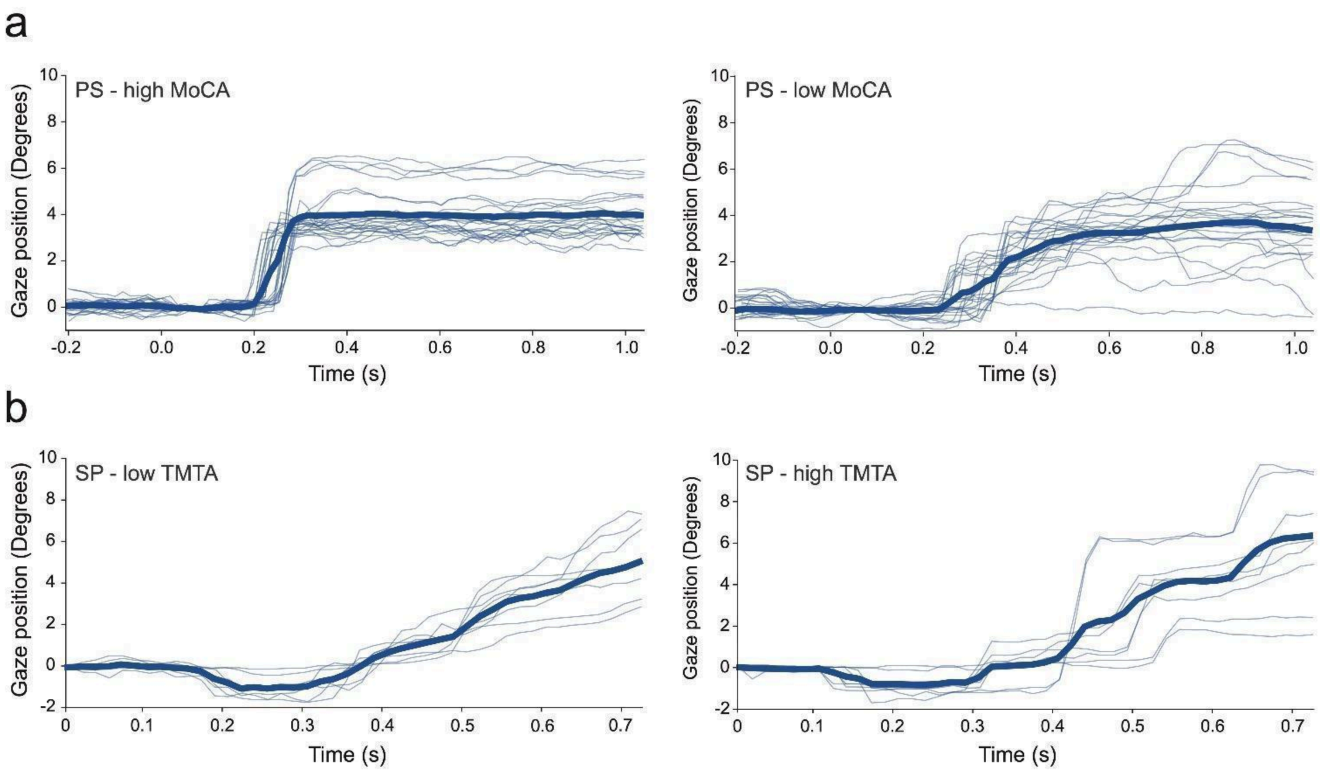

Supplementary Figure 4. Illustrated here are the gaze traces for 4 participants (light lines indicate individual trial traces, bold lines indicate the average trace). The top row (a) illustrates the gaze traces of two participants as measured during the small pro-saccade task: the left graph shows traces from a patient with a high MoCA score (30), whereas the right graph shows traces from a patient with a lower MoCA score (22); notice how the average saccade trace on the right has a much longer latency and takes longer to stabilize near the target. The bottom row (b) illustrates the gaze traces of two participants as measured during the medium velocity smooth pursuit task: the left graph shows traces from a patient with a low (good) TMTA time (29), whereas the right graph shows traces from a patient with a high TMTA time (71); notice how the average smooth pursuit trace on the right has larger amplitude catch-up saccades. PS: pro-saccade, SP: smooth pursuit

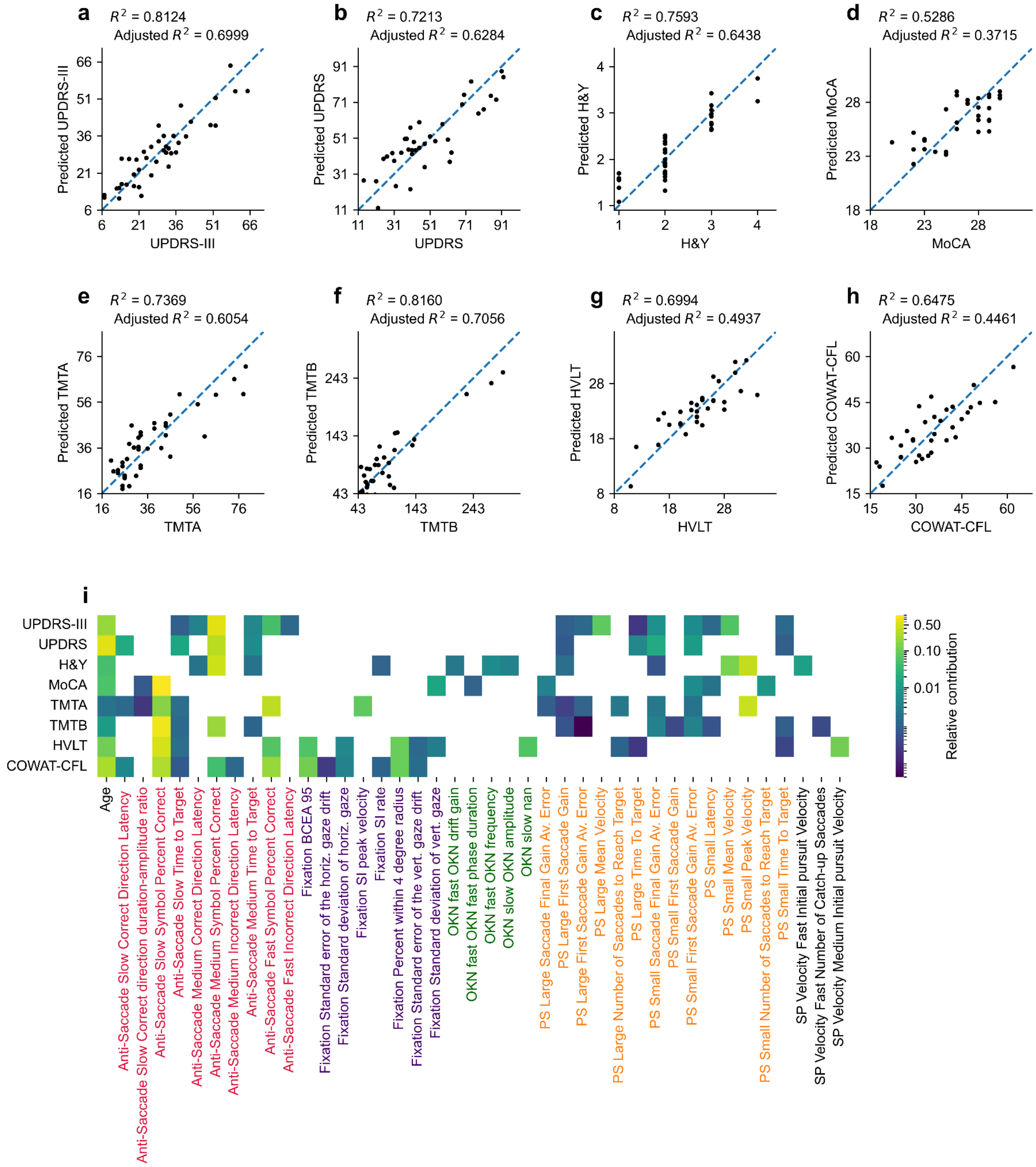

Supplementary Figure 5. (a-h) Scatterplots of the relationship between the study participants' clinical scores and the corresponding predicted value obtained by partial least squares regression analysis using the oculomotor parameters and age as predictors. **(i)** Heatmap visualization of the relative contribution (normalized absolute value of standardized regression coefficients) of each oculomotor parameter and age to each partial least squares regression predictor. Dark squares indicate lesser contributions to the model whereas lighter/yellow squares indicate greater contributions. Absent squares indicate that the parameter was not used in the final model. Eye-tracking parameter label colors indicate different oculomotor tasks. PS: pro-saccade, SP: smooth pursuit, OKN: Optokinetic nystagmus

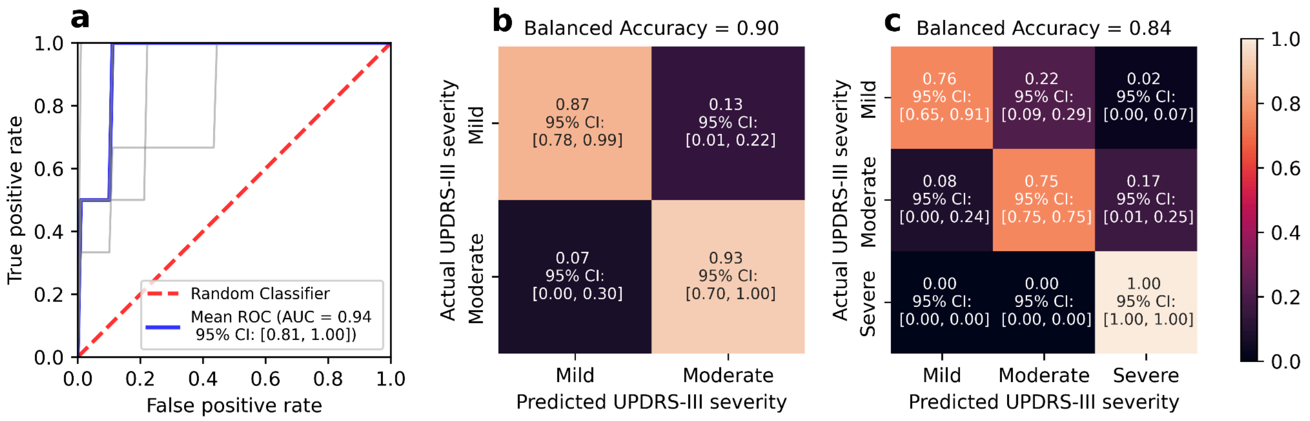

Supplementary Figure 6. Performance of the support vector classifier that includes age as an input. **(a)** Mean ROC curve for the logistic regression classifier across 5-fold cross validation. Grey lines represent each fold. **(b)** Confusion matrix for classification of mild (UPDRS-III 0-35) and moderate (UPDRS-III 36-57) Parkinson's Disease using eye tracking parameters and age. SVM classifier used Anti-Saccade Medium Symbol Percent Correct, PS Large Time To Target, PS Small Mean Velocity, PS Large Peak Velocity, Fixation Standard error of the vert. gaze drift and age with =0.000278 and C = 0.5. (c) Confusion matrix for classification of mild (UPDRS-III 0-35), moderate (UPDRS-III 36-57) and severe (UPDRS-III >57) Parkinson's Disease using eye tracking parameters and age. SVM classifier used Anti-Saccade Medium Symbol Percent Correct, Anti-Saccade Medium Correct Direction Latency, PS Large Peak Velocity, PS Large Mean Velocity, PS Large Saccade Final Gain and age as inputs with a = 0.0001 and C = 0.1984.

	UPDRS-III	UPDRS	H&Y	MOCA	HVLT	TMTA (s)	TMTB (s)	COWAT-CFL
**UPDRS-III**	1	--	--	--	--	--	--	--
**UPDRS**	**0.73 p=2.44e-07**	1	--	--	--	--	--	--
**H&Y**	**0.40 p=3.56e-03**	**0.42 p=1.08e-02**	1	--	--	--	--	--
**MOCA**	-0.31 p=8.69e-02	-0.36 p=0.159	**-0.34 p=6.24e-02**	1	--	--	--	--
**HVLT**	-0.28 p=6.78e-02	-0.20 p=0.284	-0.03 p=0.831	**0.56 p=1.54e-03**	1	--	--	--
**TMTA (s)**	**0.52 p=5.32e-04**	**0.54 p=2.22e-03**	**0.36 p=2.11e-02**	**-0.48 p=7.25e-03**	**-0.37 p=1.27e-02**	1	--	--
**TMTB (s)**	**0.48 p=1.52e-03**	**0.58 p=1.52e-03**	**0.28 p=7.33e-02**	**-0.55 p=2.22e-03**	**-0.56 p=1.35e-04**	**0.74 p=5.35e-09**	1	--
**COWAT-CFL**	-0.21 p=0.18	-0.25 p=0.18	-0.15 p=0.34	**0.48 p=8.89e-03**	0.30 p=5.88e-02	**-0.48 p=1.52e-03**	**-0.56 p=1.35e-04**	1

Supplementary Table 1. For each PD-related clinical and cognitive test scores., clinical score-clinical score correlations depicted in Figure 1j are shown. ρ, Spearman's rho. p-values for two-sided t-tests are presented after FDR correction (Benjamini-Hochberg procedure, α = 0.05). UPDRS (Unified Parkinson's Disease Rating Scale); H&Y (Hoehn and Yahr Scale); MoCA (Montreal Cognitive Assessment); TMT-A/B (Trail Making Test - A/B); HVLT (Hopkins Verbal Learning test); COWAT (Controlled Oral Word Association Test). Correlations with p ≤ 0.05 are in bold.

		UPDRS-III					UPDRS					H&Y					MOCA				
		B	Std. Error	β	t	P value	B	Std. Error	β	t	P value	B	Std. Error	β	t	P value	B	Std. Error	β	t	P value
	**Const**	30.69	175.96	0.00	0.17	0.86	50.89	32.31	0.00	1.58	0.13	2.21	1.11	0.00	2.00	0.06	**26.24**	**6.64**	**0.00**	**3.95**	**0.00**
**Anti-Saccade Slow**	**Correct Direction Latency**						11.50	27.20	0.10	0.42	0.68										
	**Correct direction duration-amplitude ratio**																-0.18	7.81	0.00	-0.02	0.98
	**Symbol Percent Correct**																**0.50**	**0.02**	**5.36**	**26.48**	**0.00**
	**Time to Target**	-5.42	24.81	-0.07	-0.22	0.83	-7.15	32.72	-0.06	-0.22	0.83										
**Anti-Saccade Medium**	**Correct Direction Latency**	-2.38	23.49	-0.02	-0.10	0.92	-3.08	52.40	-0.02	-0.06	0.95	-0.38	1.33	-0.06	-0.28	0.78					
	**Symbol Percent Correct**	**-4.41**	**0.08**	**-12.79**	**-56.86**	**0.00**	**-1.99**	**0.10**	**-2.89**	**-19.66**	**0.00**	**-0.36**	**0.00**	**-16.25**	**-103.9**	**0.00**					
	**Incorrect Direction Latency**																				
	**Time to Target**	9.91	37.00	0.09	0.27	0.79	6.19	50.67	0.04	0.12	0.90	0.43	1.32	0.07	0.33	0.75					
**Anti-Saccade Fast**	**Symbol Percent Correct**	**1.03**	**0.10**	**3.15**	**10.84**	**0.00**															
**Fixation**	**BCEA 95**																				
	**Standard error of the horiz. gaze drift**																				
	**Standard deviation of horiz. gaze**																				
	**SI peak velocity**																				
	**SI rate**											0.16	0.51	0.04	0.32	0.75					
	**Standard error of the vert. gaze drift**																				
	**Standard deviation of vert. gaze**																-0.62	1.12	-0.09	-0.56	0.58
**OKN fast**	**OKN drift gain**											0.05	0.19	0.05	0.27	0.79					
	**OKN fast phase duration**																1.02	28.56	0.01	0.04	0.97
	**OKN frequency**											**-0.03**	**0.01**	**-0.61**	**-3.53**	**0.00**					
**OKN slow**	**OKN amplitude**											**0.14**	**0.12**	**0.17**	**1.12**	**0.27**					
**PS Large**	**Saccade Final Gain Av. Error**	-3.39	41.96	-0.02	-0.08	0.94											-0.59	5.01	-0.02	-0.12	0.91
	**First Saccade Gain**	-1.17	21.16	-0.01	-0.06	0.96	-2.26	28.02	-0.01	-0.08	0.94	-0.35	1.00	-0.06	-0.35	0.73					
	**First Saccade Gain Av. Error**	5.34	49.68	0.04	0.11	0.92															
	**Mean Velocity**	**-0.62**	**0.08**	**-1.67**	**-7.39**	**0**															
	**Number of Saccades to Reach Target**																				
	**Time To Target**	2.47	54.46	0.01	0.05	0.96	7.4	56.19	0.02	0.13	0.9										
**PS Small**	**Saccade Final Gain Av. Error**	-13.55	92.53	-0.12	-0.15	0.89	15.74	93.89	0.09	0.17	0.87	-0.63	5.15	-0.1	-0.12	0.9					

		B	Std. Error	β	t	P value	B	Std. Error	β	t	P value	B	Std. Error	β	t	P value	B	Std. Error	β	t	P value
	**First Saccade Gain**																				
	**First Saccade Gain Av. Error**	15.43	91.92	0.14	0.17	0.87	-9.34	93.64	-0.06	-0.1	0.92	0.55	5.08	0.09	0.11	0.91	0.66	4.15	0.03	0.16	0.88
	**Latency**	6.85	95.77	0.02	0.07	0.94											-0.64	8.94	-0.01	-0.07	0.94
	**Mean Velocity**	**-2.23**	**0.12**	**-3.97**	**-18.86**	**0**						**0.22**	**0.01**	**6.76**	**47.49**	**0**					
	**Peak Velocity**	**-1.64**	**0.03**	**-8**	**-51.14**	**0**						**-0.19**	**0**	**-17.3**	**-137.8**	**0**					
	**Number of Saccades to Reach Target**																-1.21	5.34	-0.05	-0.23	0.82
	**Time To Target**	-7.79	88.86	-0.04	-0.09	0.93	2.46	56.84	0.01	0.04	0.97										
**SP Velocity Fast**	**Initial pursuit Velocity**											**0.12**	**0.02**	**1.56**	**6.62**	**0**					
	**Number of Catch-up Saccades**																				
	**Pursuit gain**											-0.1	0.72	-0.03	-0.14	0.89					
**SP Velocity Medium**	**Initial pursuit Velocity**																				
**SP Velocity Slow**	**Number of Catch-up Saccades**	0.38	14.88	0.01	0.03	0.98															
	**Proportion of Time in Pursuit**	3.14	179.71	0.01	0.02	0.99															

		TMTA					TMTB					HVLT					COWAT-CFL				
		B	Std. Error	β	t	P value	B	Std. Error	β	t	P value	B	Std. Error	β	t	P value	B	Std. Error	β	t	P value
	**Const**	37.80	32.59	0.00	1.16	0.26	95.55	127.63	0.00	0.75	0.46	**23.00**	**10.28**	**0.00**	**2.24**	**0.04**	35.50	20.75	0.00	1.71	0.10
**Anti-Saccade Slow**	**Correct Direction Latency**	-3.98	18.37	-0.04	-0.22	0.83											-4.19	15.71	-0.06	-0.27	0.79
	**Correct direction duration-amplitude ratio**	1.84	37.00	0.01	0.05	0.96															
	**Symbol Percent Correct**	**-2.50**	**0.07**	**-5.28**	**-34.49**	**0.00**	**-95.11**	**0.50**	**-51.49**	**-191.3**	**0.00**						**-1.21**	**0.08**	**-3.55**	**-14.59**	**0.00**
	**Time to Target**	4.96	24.00	0.05	0.21	0.84	-37.93	95.10	-0.09	-0.40	0.69						-1.34	23.23	-0.02	-0.06	0.96
**Anti-Saccade Medium**	**Correct Direction Latency**											-0.32	7.59	-0.01	-0.04	0.97					
	**Symbol Percent Correct**						**24.45**	**0.32**	**13.63**	**75.58**	**0.00**	**1.14**	**0.03**	**7.29**	**35.91**	**0.00**	**-1.34**	**0.10**	**-4.01**	**-13.85**	**0.00**
	**Incorrect Direction Latency**																-3.22	14.45	-0.04	-0.22	0.83
	**Time to Target**						42.87	113.23	0.08	0.38	0.71						-2.54	26.45	-0.02	-0.10	0.92
**Anti-Saccade Fast**	**Symbol Percent Correct**	**-4.50**	**0.07**	**-11.24**	**-65.00**	**0.00**											**4.52**	**0.07**	**16.80**	**64.86**	**0.00**
**Fixation**	**BCEA 95**											**4.58**	**0.79**	**2.92**	**5.78**	**0.00**	**-4.39**	**1.44**	**-1.27**	**-3.04**	**0.01**
	**Standard error of the horiz. gaze drift**																-2.11	58.45	-0.01	-0.04	0.97
	**Standard deviation of horiz. gaze**											**-4.67**	**9.07**	**-0.18**	**-0.52**	**0.61**	1.71	18.61	0.03	0.09	0.93
	**SI peak velocity**	**0.62**	**0.03**	**2.61**	**21.11**	**0.00**															
	**SI rate**																1.64	8.59	0.03	0.19	0.85

	**Standard error of the vert. gaze drift**											-2.52	21.91	-0.05	-0.12	0.91	1.64	23.41	0.02	0.07	0.95
	**Standard deviation of vert. gaze**											1.03	4.68	0.07	0.22	0.83					
**OKN fast**	**OKN drift gain**																				
	**OKN fast phase duration**																				
	**OKN frequency**																				
**OKN slow**	**OKN amplitude**											**0.98**	**0.08**	**2.12**	**12.90**	**0.00**					
**PS Large**	**Saccade Final Gain Av. Error**	4.94	39.18	0.03	0.13	0.90															
	**First Saccade Gain**	1.26	23.24	0.01	0.05	0.96	-14.38	100.29	-0.03	-0.14	0.89										
	**First Saccade Gain Av. Error**	-6.04	52.67	-0.03	-0.12	0.91	1.14	118.66	0	0.01	0.99										
	**Mean Velocity**																				
	**Number of Saccades to Reach Target**	7.96	14.73	0.08	0.54	0.59						-1.68	5.24	-0.05	-0.32	0.75					
	**Time To Target**											-0.33	20.16	0	-0.02	0.99					
**PS Small**	**Saccade Final Gain Av. Error**	9.3	86.06	0.07	0.11	0.92	-126.6	396.7	-0.26	-0.32	0.75										
	**First Saccade Gain**						9.03	78.73	0.03	0.12	0.91										
	**First Saccade Gain Av. Error**	-7.67	85.01	-0.06	-0.09	0.93	104.98	362.05	0.23	0.29	0.78										
	**Latency**						56.76	256.23	0.04	0.22	0.83										
	**Mean Velocity**																				
	**Peak Velocity**	**-3.41**	**0.03**	**-13.36**	**-114.0**	**0**															
	**Number of Saccades to Reach Target**																				
	**Time To Target**						-67.85	238.43	-0.06	-0.29	0.78	0.44	16.2	0	0.03	0.98					
**SP Velocity Fast**	**Initial pursuit Velocity**																				
	**Number of Catch-up Saccades**						5.3	17.6	0.03	0.3	0.77										
	**Pursuit gain**																				
**SP Velocity Medium**	**Initial pursuit Velocity**											**3.51**	**0.15**	**3.83**	**23.3**	**0**					
**SP Velocity Slow**	**Number of Catch-up Saccades**																				
	**Proportion of Time in Pursuit**																				

Supplementary Table 2. Multiple regression tables for UPDRS-III, UPDRS, H&Y, MoCA, TMTA, TMTB, HVLT, and COWAT-CFL. Regression coefficients (B), standard error, standardized regression coefficients (ß), t-statistic and associated p-value (two-sided test) for each oculomotor parameter. PS: pro-saccade, SP: smooth pursuit, OKN: Optokinetic nystagmus. Correlations with $p \leq 0.05$ are in bold.
